# DNMTs and SETDB1 function as co-repressors in MAX-mediated repression of germ cell–related genes in mouse embryonic stem cells

**DOI:** 10.1371/journal.pone.0205969

**Published:** 2018-11-07

**Authors:** Daiki Tatsumi, Yohei Hayashi, Mai Endo, Hisato Kobayashi, Takumi Yoshioka, Kohei Kiso, Shinichiro Kanno, Yuji Nakai, Ikuma Maeda, Kentaro Mochizuki, Makoto Tachibana, Haruhiko Koseki, Akihiko Okuda, Akira Yasui, Tomohiro Kono, Yasuhisa Matsui

**Affiliations:** 1 Cell Resource Center for Biomedical Research, Institute of Development, Aging and Cancer (IDAC), Tohoku University, Sendai, Miyagi, Japan; 2 Graduate School of Life Sciences, Tohoku University, Sendai, Miyagi, Japan; 3 The Japan Agency for Medical Research and Development-Core Research for Evolutional Science and Technology (AMED-CREST), Chuo-ku, Tokyo, Japan; 4 NODAI Genome Research Center, Tokyo University of Agriculture, Setagaya-ku, Tokyo, Japan; 5 Department of Bioscience, Tokyo University of Agriculture, Setagaya-ku, Tokyo, Japan; 6 Tohoku University School of Medicine, Sendai, Miyagi, Japan; 7 Division of Dynamic Proteome in Cancer and Aging, Institute of Development, Aging and Cancer, Tohoku University, Sendai, Miyagi, Japan; 8 Institute for Food Sciences, Hirosaki University, Hirosaki, Aomori, Japan; 9 Center for Environmental Conservation and Research Safety, Tohoku University, Sendai, Miyagi, Japan; 10 Department of Enzyme Chemistry, Institute for Enzyme Research, Tokushima University, Shinkura-cho, Tokushima, Japan; 11 Laboratory for Developmental Genetics, RIKEN Center for Integrative Medical Sciences, Yokohama, Kanagawa, Japan; 12 Core Research for Evolutional Science and Technology, Yokohama, Kanagawa, Japan; 13 Division of Developmental Biology, Research Center for Genomic Medicine, Saitama Medical University, Yamane Hidaka, Saitama, Japan; 14 Center for Regulatory Epigenome and Diseases, Tohoku University School of Medicine, Sendai, Miyagi, Japan; University of Bonn, Institute of Experimental Hematology and Transfusion Medicine, GERMANY

## Abstract

In embryonic stem cells (ESCs), the expression of development-related genes, including germ cell–related genes, is globally repressed. The transcription factor MAX represses germ cell–related gene expression in ESCs via PCGF6-polycomb repressive complex 1 (PRC1), which consists of several epigenetic factors. However, we predicted that MAX represses germ cell–related gene expression through several additional mechanisms because PCGF6-PRC1 regulates the expression of only a subset of genes repressed by MAX. Here, we report that MAX associated with DNA methyltransferases (DNMTs) and the histone methyltransferase SETDB1 cooperatively control germ cell–related gene expression in ESCs. Both DNA methylation and histone H3 lysine 9 tri-methylation of the promoter regions of several germ cell–related genes were not affected by knockout of the PRC1 components, indicating that the MAX-DNMT and MAX-SETDB1 pathways are independent of the PCGF6-PRC1 pathway. Our findings provide insights into our understanding of MAX-based repressive mechanisms of germ cell–related genes in ESCs.

## Introduction

Embryonic stem cells (ESCs) derived from the inner cell mass of blastocysts maintain a pluripotent state via the global repression of development-related genes [1], which is dependent upon multiple epigenetic modifications controlled by several multiprotein complexes. We previously explored genes involved in the repression of germ-cell related genes in ESCs by an RNA interference screen. The expression of a germ cell-specific *Vasa*-Red fluorescent protein (RFP) reporter genes was monitored after knockdown (KD) of 864 transcription factor genes expressed in ESCs, resulting in the identification of candidate genes, including *Max* and *L3mbtl2*, which repress the *Vasa* reporter expression in ESCs [2]. The transcriptome profile of *Max*-KD ESCs, examined by microarray analysis, revealed the comprehensive repression of germ cell-related genes by MAX. We also showed that the euchromatic histone-lysine N-methyltransferases G9A and GLP, which together catalyze the di-methylation of histone H3 lysine 9 (H3K9me2), are also required for the repression of MAX-associated germ cell–related genes in ESCs [2]. In addition, *Max*-null ESCs exhibit a meiosis-like state (i.e., cytologic changes in germ cells at the leptotene and zygotene stages of meiosis) [3,4].

MAX, L3MBTL2 and G9A are components of Polycomb repressive complex (PRC)1. Polycomb group proteins (PcGs) constitute chromatin-modifying complexes that function as transcriptional repressors of development-related genes in ESCs [5,6,7]. Two major groups of PcGs, PRC1 and PRC2, function together and modify histones [5,6]. PRC2 catalyzes H3K27me3, while PRC1 is involved in additional modifications. PRC1 contains five core proteins, each of which constitutes different protein families including CBX (CBX2/4/6/7/8, binding factors to H3K27me3), RING1 (RING1A/B, responsible E3 ubiquitin ligases for H2AK119Ub1), PHC (PHC1/2/3), PCGF (PCGF1-6, polycomb group ring finger), and RYBP/ YAF2. Different combinations of each of the five components can generate diverse PRC1 complexes [7,8]. In a previous report, six types of PRC1-family complexes were defined and classified according to the diversity of PCGF factors (PCGF1-PCGF6), which directly associate with RING1A/B proteins [9]. One of these subtypes, PCGF6-containing PRC1 (PCGF6-PRC1), was identified as a complex consisting of several transcription factors (E2F6, MAX, MGA, and L3MBTL2) and epigenetic enzymes (HDAC1, HDAC2, and G9A) [10–12]. Elevated expression of germ cell–related genes, including *Ddx4*, in *Pcgf6*- and *L3mbtl2*-knockout (KO) ESCs indicates that PCGF6-PRC1 suppresses the expression of germ cell–related genes in ESCs [13]. In addition, in female primordial germ cell (PGC) development, RING1B is required for normal development to prevent premature entry into meiotic prophase [14].

Although MAX-containing PCGF6-PRC1 represses the expression of germ cell–related genes in ESCs as described above, MAX also likely interacts with epigenetic regulators other than PCGF6-PRC1, but this has not been confirmed. In this study, we examined whether MAX repressed germ cell-related genes by other mechanisms than PCGF6-PRC1, and found that MAX represses germ cell–related genes in ESCs through DNA methyltransferases (DNMTs) and a H3K9 methyltransferase, SETDB1 in addition to PCGF6-PRC1. Our data suggest that MAX interacts with various epigenetic regulators to control the expression of germ cell–related genes in ESCs, which may be crucial for maintenance of these cells.

## Materials and methods

### ESC culture

VV3 [2], *Max*-null [4], *Dnmt1*, *Dnmt3a*, and *Dnmt3b* triple-knockout (TKO) ESCs (*Dnmts*-TKO ESCs; *Dnmt1*^–/–^*Dnmt3a*^–/–^*Dnmt3b*^–/–^) [15], *Ring1b* and *Ring1a* (a paralog of *Ring1b*) double-knockout (*Ring1a/b*-DKO; *Ring1a*^–/–^*Ring1b*^fl/fl^*Rosa26*::*CreERT2*) [16], and *G9a*-KO (*G9a*^–/–^) ESCs [17] were cultured as described previously. All ESCs were cultured in conventional ES medium with serum and leukemia inhibitory factor (LIF). *Max*-null and *Dnmts*-TKO ESCs were cultured without feeder cells. VV3 ESCs were cultured on STO feeder cells inactivated with mitomycin C, whereas *Ring1a/b*-DKO and *G9a*-KO ESCs were cultured on inactivated mouse embryonic fibroblasts. In *Max*-null ESCs, both alleles of the *Max* gene are disrupted and *Max* cDNA was introduced into the *ROSA26* locus under the control of a tetracycline-off system [4]. In *Ring1a/b*-DKO ESCs, both alleles of the *Ring1a* gene are disrupted and the *Ring1b* gene are floxed, and *Ring1b* could be conditionally deleted by 4-hydroxy tamoxifen (OHT) treatment [16]. For KO of *Max* and *Ring1b*, cells were treated for 3 days with doxycycline (Dox) (1 μg/ml) or 4-hydroxytamoxifen (4OHT) (800 nM), respectively.

### Transfection of siRNAs

For KD assays, cells were transfected with siRNAs using Lipofectamine RNAiMAX (Invitrogen) by the reverse method a 24-well plate according to the manufacturer’s instructions. Briefly, Lipofectamine RNAiMAX (2 μl) and siRNA (16–48 pmol) were diluted with 100 μl of OptiMEM (gibco) and incubated for 20 min. An aliquot of 50,000 ESCs in 500 μl of ES medium (Glasgow’s Modified Eagle’s Medium [GMEM, Wako] supplemented with 10% fetal bovine serum [FBS], 0.1 mM nonessential amino acids [gibco], 1 mM sodium pyruvate [gibco], 100 μM ß-mercaptoethanol, 1,000 U/ml LIF [Millipore]) was added to each Lipofectamine/siRNA sample, mixed, and plated into separate wells of a 24-well plate. The cells were incubated for 24 h and fed ES medium. All siRNAs were designed by Qiagen. The following siRNAs were used in this study: Mm_Max_5, Mm_Setdb1_5, Mm_Hdac1_1, Mm_Hdac2_5, Mm_Atf7ip_3, Mm_L3mbtl2_4, and AllStars (as a negative control siRNA; Qiagen).

### Conventional bisulfite sequencing

Bisulfite sequencing analysis by Sanger sequencing was carried out as described previously [18]. *Max*-KD VV3 ESCs with a *Vasa*-Venus reporter or control ESCs (ESCs transfected with AllStars negative control siRNA) were cultured for approximately 72 h and sorted using an S3e cell sorter (Bio-Rad). *Max*-KD VV3 ESCs were purified based on *Vasa*::Venus-positivity, and control ESCs were purified based on *Vasa*::Venus-negativity. Genomic DNA was extracted from both cell types using a Qiagen DNeasy blood & tissue kit or Qiagen All-prep DNA/RNA micro kit and converted with sodium bisulfite using an EZ DNA methylation-direct kit (Zymo Research) according to the manufacturer’s instructions. The targeted regions were amplified from bisulfite-converted DNAs using BIOTAQ HS DNA Polymerase (Bioline). The sequences of the PCR primers used for this assay are shown in S1 Table. The PCR products were cloned into respective pGEM-T easy vectors (Promega) and sequenced using a BigDye Terminator v1.1 cycle sequencing kit (Applied Biosystems).

### Targeted methylome sequencing (TMS)

#### DNA preparation for TMS

DNA was isolated from sorted *Max*-KD VV3 ESCs and control ESCs using a DNeasy blood & tissue kit (Qiagen); 1 μg of DNA was dissolved in 130 μl of 10 mM Tris-HCl (pH 8.0) and sheared using an S220 focused ultrasonicator (Covaris) to yield 500-bp fragments. An AMPure XP system (Agilent Technologies) was used to purify the fragmented DNA as follows. Sheared DNA (130 μl) was mixed with 1.8 volumes (234 μl) of AMPure XP reagent and allowed to stand for 15 min at room temperature. The beads were collected using a magnetic stand, the supernatant was removed, and pelleted beads were rinsed with 70% ethanol and dried by incubation at 37°C for 5 min. DNA was then eluted from the beads using 20 μl of RNase-free water. The eluted DNA was dried under vacuum and then dissolved in 7 μl of RNase-free water.

#### Target enrichment for TMS

A SureSelect Mouse Methyl-Seq kit (Agilent Technologies) was used for target enrichment by liquid-phase hybridization capture [19]. The probe set used in this study is designed by Agilent Technologies to comprehensively detect promoters, enhancers, and gene bodies. Genomic DNA (7 μl) fragmented and purified as described above was supplemented with 3 μl of formamide (biochemistry grade; Wako) and overlaid with 80 μl of mineral oil (Sigma-Aldrich). The DNA was then completely denatured by incubating at 99°C for 10 min; the sample was then cooled to and maintained at 65°C for at least 5 min before adding the following reagents. Hybridization buffer and capture probe mix were prepared according to the manufacturer’s protocol, and they were each overlaid with 80 μl of mineral oil and incubated at 65°C for 10 min. The two solutions were then combined and mixed thoroughly by pipetting. The combined solution was transferred to a tube containing the denatured input DNA (maintained at 65°C as described above), and the solution was thoroughly mixed by pipetting. The sample was incubated at 65°C for 24 h to allow for probe/target hybridization. A 50-μl volume of well-suspended DynaBeads MyOne streptavidin T1 solution (Life Technologies) was placed in a 1.5-ml tube, and the beads were washed twice with 200 μl of binding buffer. The hybridization reaction mixture, supplemented with 200 μl of binding buffer, was then added to the pelleted beads and thoroughly mixed. After incubation at room temperature for 30 min with agitation, the beads were collected using a magnetic stand and washed with 500 μl of wash buffer 1, subjected to three rounds of washing and resuspension in pre-warmed buffer 2, then incubated at 65°C for 10 min. After removing the washing solution, the enriched DNA was eluted by incubating the beads in 20 μl of elution buffer at room temperature for 20 min. The eluate was immediately subjected to bisulfite treatment.

#### Bisulfite treatment for TMS

An EZ DNA methylation-gold kit (Zymo Research) was used for bisulfite treatment of target-enriched DNA according to the manufacturer’s instructions. Enriched DNA solution (20 μl) was mixed with 130 μl of freshly prepared CT conversion reagent, and the mixture was incubated at 64°C for 2.5 h. The 10-min incubation step at 98°C was omitted because the target-enriched DNA was already denatured. After purification and desulfonation, bisulfite-treated DNA was eluted with 20 μl of M-elution buffer.

#### TMS library construction and illumina sequencing

We used bisulfite-treated DNA for library preparation according to the PBAT protocol [20] (also available from http://crest-ihec.jp/english/epigenome/index.html), except for use of the primers described below. The primer used for first-strand synthesis was 5’-biotin ACA CTC TTT CCC TAC ACG ACG CTC TTC CGA TCT WWW WNN NN-3’ (W 1/4 A or T). The indexed primer used for second-strand synthesis was 5’-CAA GCA GAA GAC GGC ATA CGA GAT XXX XXX GTA AAA CGA CGG CCA GCA GGA AAC AGC TAT GAC WWW WNN NN-3’, where XXX XXX represents the index sequence of each primer. The constructed TMS libraries were sequenced as previously described [20–23] using an HiSeq2500 system (illumina).

#### TMS alignment and statistical analysis

TMS reads were aligned to the mouse genome (mm10; Genome Reference Consortium Mouse Build 38) using the Bismark tool (v.0.10.0; http://www.bioinformatics.babraham.ac.uk/projects/bismark/), with the following specific options: q n 2 –l 93 –pbat. MOABS module [24] was applied to detect differentially methylated regions (DMRs) from TMS reads of *Max*-KD VV3 ESCs and control ESCs (2 biological replicates). The MOABS pipeline calls DMR candidates by 3 different methods (M1, M2, and M3). In our case, M2 method identified the largest numbers of DMRs (no false positives). Since M2-called DMRs include almost M1- and M3-called DMRs, we used the M2-called DMRs for following analyses. In MOABS M2 method (according to the credible methylation difference metric), DMRs were defined as minC (minimum coverage of targeted regions) ≥ 10, maxDist (maximum distance of DMRs from genes) 300 base pairs, cMethDif (credible Methylation Difference Cutoff) > 0.2. cMethDif > 0.2 means that only the regions where the DNA methylation levels in *Max*-KD ESCs show differences larger than 20% compared with those in control ESCs identify as DMRs. Motifs in DMRs were identified using the motif call tool, findMotifs.pl, from HOMER (http://homer.salk.edu/homer/motif/).

### RNA preparation and real-time PCR

Total RNA isolated from cells was purified using an RNeasy Plus Mini kit (Qiagen) or RNeasy Micro kit (Qiagen) according to the manufacturer’s instructions. RNAs were reverse-transcribed using SuperScript III (Invitrogen) and random primers (Promega). Gene expression was quantified using SYBR Green master mix (Applied Biosystems) with the primers shown in S1 Table. PCR signals were detected using CFX Connect (Bio-Rad). *Arbp* was used as an internal control.

### Microarray analysis of *L3mbtl2*-KD ESCs

Microarray analyses were carried out as described previously [2]. VV3 ESCs were transfected with non-silencing negative control siRNA (AllStars) or siRNA against the *L3mbtl2* gene. Vasa-positive cells were purified using fluorescence-activated cell sorting (FACS). Total RNA (100 ng) was isolated and purified using an RNeasy micro kit (Qiagen). The quality and quantity of purified total RNA were verified by Agilent 2100 Bioanalyzer (Agilent) and NanoDrop ND-1000 (Thermo Fischer Scientific), respectively. DNA microarray analysis was carried out according to manufacturer’s instruction. In brief, cyanine3-labelled cRNA was obtained from 100 ng of purified total RNA using a Low Input Quick Amp Labeling kit (Agilent). The cRNA was purified, fragmented and then hybridized to an Agilent Whole Mouse Genome Oligo DNA Microarray kit, Ver 2.0 (Agilent) containing over 44,000 probes for mouse genes. Following hybridization at 65°C for 17h, the arrays were washed and fluorescence signals were scanned using an Agilent DNA microarray scanner. Agilent Feature Extraction software was used to reduce the array images to the intensity of each probe (TXT files). Each cell type was analyzed in four biological replicates. All the microarray data have been deposited in the National Center for Biotechnology Information (NCBI) Gene Expression Omnibus (http://www.ncbi.nlm.nih.gov/geo/, GEO Series accession number GSE102610).

### Microarray analysis of *G9a*-KO ESCs

Total RNAs of *G9a*-KO ESCs and control ESCs (TT2) were isolated and purified using an RNeasy mini kit (Qiagen). The quality and quantity of purified total RNA were verified by agarose gel electrophoresis and spectrophotometry, respectively. DNA microarray analysis was carried out according to manufacturer’s instruction. In brief, biotinylated cRNA was obtained from 200 ng of purified total RNA using a GeneChip 3’ IVT Express Kit (Affymetrix). The cRNA was purified, fragmented and then hybridized to an Affymetrix Mouse Genome 430 2.0 Array containing over 45,000 probe sets for mouse genes. Following hybridization at 45°C for 16h, the arrays were washed and labeled with phycoerythrin. Fluorescence signals were scanned using the Affymetrix GeneChip System. Affymetrix GeneChip Command Console software was used to reduce the array images to the intensity of each probe (CEL files). All the microarray data are MIAME compliant and have been deposited in a MIAME compliant database, the National Center for Biotechnology Information (NCBI) Gene Expression Omnibus (http://www.ncbi.nlm.nih.gov/geo/, GEO Series accession number GSE102423), as detailed on the FGED Society website (http://fged.org).

### Transcriptome data analysis

GeneSpring (version 12.6, Tomy Digital Biology) was used for the identification of differentially-expressed genes (DEGs), statistical analysis, gene ontology (GO) analysis and description of Venn diagrams for microarray data. Seventy-five percentile shift was used to obtain normalized intensities for every feature on the array. We calculated the ratio of intensity in KO or KD samples to the intensity in the respective control samples as expression change. DEGs were determined as the genes in which the expression change of at least one probe is more than the expected fold change. Multiple testing corrections were performed using the Benjamini-Hochberg false-discovery rate correction. For the microarray data of *Max*-KD ESCs (GSE45181) [2] and *L3mbtl2*-KD ESCs obtained in our previous and this studies, respectively, gene expression profiles of *Vasa*-positive cells isolated from VV3 ESCs transfected with siRNAs for *Max* or *L3mbtl2* were compared to those of VV3 ESCs transfected with non-silencing negative control siRNA (AllStars). For the microarray data of *G9a*-KO ESCs and *Dnmts*-TKO ESCs (GSE20177) [25] obtained in this study and by another group, respectively, gene expression profiles of wild-type (WT) and KO ESCs were compared. For the microarray data of *Ring1a/b*-DKO ESCs (GSE10573) [16], and *Setdb1*-KO ESCs (GSE28593) [26] obtained by other groups, gene expression profiles of conditional KO ESCs with OHT were compared with those of control ESCs without OHT. For RNA-seq analysis, RNA-seq datasets for *Pcgf6*-KO ESCs (GSE84480) [13], *Dnmt1*-cKO E13.5 PGCs (GSE74938) [27] and *Setdb1*-cKO E13.5 PGCs (GSE60377) [28] published by other groups were downloaded from GEO. The RNA-seq reads were aligned to the mouse reference genome (UCSC mm9 and RefSeq) using TopHat (ver. 2.0.8) [29]. Cufflinks (ver. 2.0.10) was used to estimate gene expression levels on the basis of fragments per kilobase of exon model per million mapped fragments [30]. For *Pcgf6*-KO ESCs, gene expression profiles of conditional KO ESCs with OHT were compared with those of control ESCs without OHT. For *Dnmt1*-cKO and *Setdb1*-cKO E13.5 PGCs, gene expression profiles of WT and KO PGCs were compared. The details of each sample can be accessed via these GEO accession numbers.

### Immunoprecipitation and Western blotting

Anti-MAX antibody (Santa Cruz, sc-197x) (2 μg) were bound to Protein G Dynabeads (Invitrogen 10007D) in PBS for 2 h at 4°C. After washing with Cross-linking buffer (50 mM phosphate buffer [pH 8.0], 20 mM triethanolamine [pH 8.0]), the precipitate was incubated in Cross-linking buffer with 5 mM Dimethyl pimelimidate dihydrochloride (SIGMA) for 30 min at room temperature. After washing with PBS, the precipitate was incubated in blocking buffer (50 mM phosphate buffer [pH 8.0], 10 mM triethanolamine [pH 8.0]) for 1 h at 4°C. After washing with PBS and 0.1 M glycine (pH 3.5), the precipitate was suspended with PBS and used for immunoprecipitation. VV3 ESCs were harvested and suspended with Buffer A (10 mM Hepes-NaOH [pH 7.9], 10 mM KCl, 1.5 mM MgCl_2_, 1× cOmplete protease inhibitor cocktail [Roche]). The cell suspension was homogenized using a 120 Vac Overhead Stirrer (Wheaton), and the nuclear fraction was extracted by centrifugation. Collected nuclei were washed with Buffer A and re-suspended with Buffer B (20 mM Hepes-NaOH [pH 7.9], 350 mM NaCl, 1.5 mM MgCl_2_, 0.2 mM EDTA, 0.1% NP-40, 10% glycerol, 0.5 mM DTT, 1× protease inhibitor). The nuclear suspension was centrifuged, and the supernatant was incubated with antibody-bound beads overnight at 4°C. After three washes with Buffer B, the precipitate was eluted with 20 μl of 0.1 M glycine (pH 3.5). The elution was performed twice for the Western blotting using anti-DNMT1 and anti-DNMT3B antibodies (S4A Fig). The eluted proteins were used for Western blotting as described previously [2]. The antibodies used for these assays are listed in S2 Table. Anti-DNMT3L antibody were kindly provided from Dr. Keisuke Nimura [31].

### Chromatin immunoprecipitation (ChIP) by cross-linking

For ChIP of MAX, RING1B, and SETDB1, ChIP-qPCR experiments were carried out as described previously [32], with some modifications. In brief, 1–5 μg of antibodies were bound to Dynabeads Protein G (Invitrogen) overnight at 4°C. Cells were fixed using the ethylene glycol bis (succinimidyl succinate)/formaldehyde dual cross-linking method, as described previously [33]. The cross-linked cells were washed, collected in pellets by centrifugation, and flash frozen with liquid nitrogen. The cells were then lysed in SDS lysis buffer (1% SDS, 50 mM Tris-HCl [pH 8.0], 10 mM EDTA), and genomic DNA was sheared by ultrasonic fragmentation using a Bioruptor UCD-300 over 12 medium cycles (Cosmo Bio). After centrifugation, the cleared lysates were incubated with antibody-bound Dynabeads overnight at 4°C. The beads were then washed, and chromatin was eluted using ChIP direct elution buffer (10 mM Tris-HCl [pH 8.0], 300 mM NaCl, 5 mM EDTA, 0.5% SDS). The eluted chromatin was subjected to reverse cross-linking with 10% SDS for 8 h at 65°C. DNA was purified using a Qiagen PCR purification kit and analyzed via real-time PCR using Power SYBR Green PCR master mix (Applied Biosystems) and primers that spanned the TSSs of the genes of interest. The primer sequences and antibodies used for these assays are listed in S1 and S2 Tables, respectively.

### ChIP using micrococcal nuclease (MNase)

For ChIP of H3K9me2 and H3K9me3, cells were fixed with 1.0% formaldehyde for 10 min at room temperature, after which glycine was added to the medium to a final concentration of 125 mM. The cells were then incubated in 1% NP-40, 50 mM Hepes/NaOH (pH 7.5), 10 mM KCl, 15 mM MgCl_2_, and 1× cOmplete protease inhibitor cocktail (Roche) for 15 min at 4°C. After centrifugation, the cells were stored at –80°C until analyzed. The thawed lysates were subjected to MNase treatment with MNase mixture (100U MNase [New England BioLabs], 15 mM Hepes/NaOH pH7.5, 60 mM KCl, 15 mM NaCl, 0.32 mM sucrose, 3 mM CaCl_2_, 1× cOmplete protease inhibitor cocktail [Roche]) for 20 min at 37°C in order to obtain oligo- and mononucleosomes. Subsequent immunoprecipitation and detection processes were performed using the ChIP cross-linking method described above.

### Nuclear extracts preparation and fractionation

Crude nuclear fractions were prepared from mouse VV3 ESCs by homogenization in SHE buffer (10 mM HEPES pH 7.4, 0.21 M mannitol, 0.07 M sucrose, 0.1 M EDTA, 0.1 M EGTA, 0.15 mM spermine, 0.75 mM spermidine). The supernatant obtained by centrifugation (900 g, 10 min) was re-centrifuged (2000 g, 10 min). The obtained pellets (crude nuclear) were suspended in nuclear extraction buffer (50 mM HEPES pH 7.4, 0.3 M NaCl, 0.2% NP40, 1× cOmplete protease inhibitor cocktail [Roche]) and sonicated for 15sec. The suspension was centrifuged again (12,000g, 10 min). The supernatant (nuclear extract) was dialyzed against buffer A (50 mM Tris-HCl pH7.5, 50 mM NaCl, 0.2% NP40). Crude nuclear extracts were separated into four fractions (A—D) by step-gradient (0.05, 0.3, 0.6, 1.0 M NaCl) on HiTrapTM Heparin HP column (GE healthcare, HPLC system: Bio-Rad Biologic HR workstation). Each fraction was concentrated and desalted by centrifugal a filter unit Amicon ultra-4-10k (Millipore) and further separated into four fractions (I—IV) by step-gradient (0.05, 0.3, 0.6, 1.0 M NaCl) on HiTrapTM Q HP column (GE healthcare). Each fraction was concentrated and desalted by a centrifugal filter unit Amicon ultra-4-10k (Millipore).

### Immunoprecipitation of each fraction

Subsequent immunoprecipitation of each fraction was performed using the immunoprecipitation method described above with some modifications. The nuclear fractions were incubated with anti-MAX antibody-bound Protein G Dynabeads (Invitrogen) for 12 h in the presence of Benzonase nuclease (Novagen) at 4°C. After washing three times with washing buffer (0.15 M NaCl, 0.1% NP-40, 50 mM HEPES [pH 7.4]), the antibody-Protein G beads were suspended in SDS-PAGE sample buffer. After boiling for 5min, the samples were resolved by SDS-PAGE and probed by western blotting with indicated antibodies.

## Results

### Contribution of PCGF6-PRC1 to MAX-mediated gene repression

To identify additional epigenetic factors associated with MAX, but not included in PCGF6-PRC1, for repression of germ cell-related genes, we used *Max*-null ESCs for RT-qPCR and ChIP-qPCR analyses because we can easily obtain a large number of *Max*-depleted cells compared with *Max*-KD ESCs, and do not need feeder cells and transfection of siRNAs for the *Max*-null ESCs. In agreement with our previous results [3,4], Dox-induced *Max* KO in ESCs led to the reduced expression of MAX protein and significant upregulation of *Ddx4* (also known as *Mvh*, mouse *Vasa* homologue), *Dazl*, *Stra8*, and *Sycp3* (genes defined as the late PGC markers in this manuscript) expression (S1A and S1B Fig). We also confirmed that MAX was enriched in the transcription start sites (TSSs) of these genes and that enrichment declined dramatically following Dox treatment, whereas only slight enrichment was observed in the TSS of the hemoglobin-ß gene (*Hbb-b1*), which is not a target of MAX, with or without Dox (S1C Fig).

**Fig 1:**
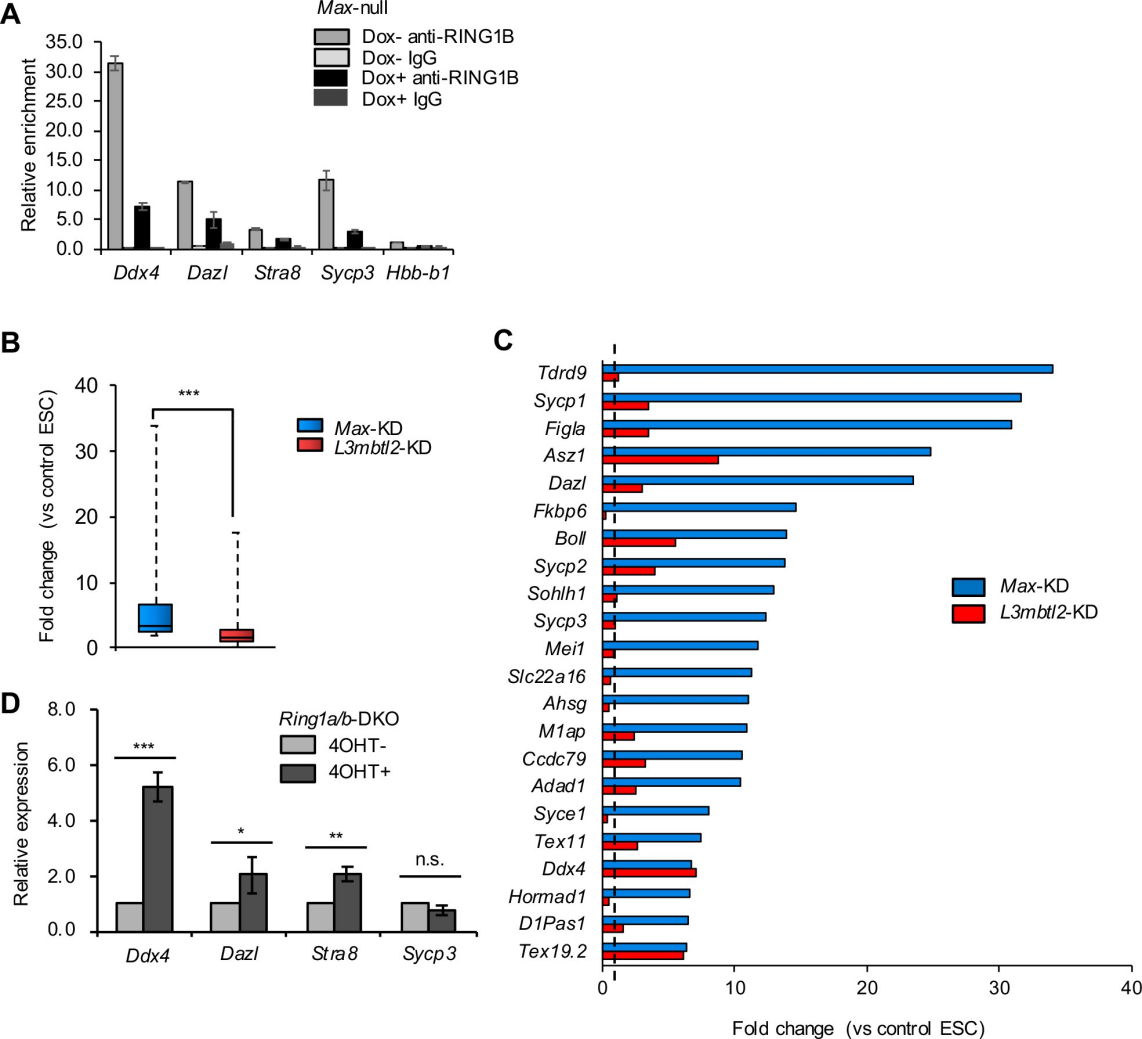
Contribution of PCGF6-PRC1 to MAX-mediated gene repression. (A) ChIP-qPCR analyses of *Max*-null ESCs (Dox+) and control ESCs (Dox−) using anti-RING1B antibody or control IgG in the promoter region of the late PGC markers and of hemoglobin β (*Hbb-b1*) as a negative control of MAX localization. Relative ratio of immunoprecipitated chromatin to input chromatin (% of input) was determined using real-time PCR. Data are presented as relative % of input normalized against % of input using anti-RING1B antibody in *Hbb-b1* in control ESCs. The bar graph represents mean ± standard error of the mean (SEM) of two independent experiments. (B) Expression changes of 85 germ cell–related up-regulated genes (*n* = 3, > 2-fold change, one-way analysis of variance [ANOVA] *P* < 0.05) in *Max*-KD or *L3mbtl2*-KD ESCs compared with control ESCs based on our microarray data (GSE45181) [2]. Fold-change in the expression is shown by a box-whisker plot. The lines inside the boxes show the median. The whiskers indicate the minimum and maximum. ***P < 0.001 (Mann-Whitney *U*-test). (C) Fold-change in the expression of significantly up-regulated germ cell–related genes (top 22 of 85 germ cell-related upregulated genes in Fig 1B) in *Max*-KD and *L3mbtl2*-KD ESCs. The expression in control ESCs was set as 1.0 (dotted line). (D) Relative expression of the late PGC markers in *Ring1a/b*-DKO ESCs (4OHT+) determined by qRT-PCR. The expression in control ESCs (4OHT−) was set as 1.0. Values are plotted as mean ± SEM of 3 biological replicates. n.s.: not significant, *P < 0.05, **P < 0.01, ***P < 0.001 (Student’s *t*-test).

We then confirmed involvement of MAX in PCGF6-PRC1-dependent repression of germ cell–related genes, and evaluated the MAX dependency of the localization of RING1B (a catalytic subunit of PRC1) to germ cell–related genes. RING1B was enriched in the TSSs of the late PGC markers compared with the TSS of *Hbb-b1*, and *Max* KO decreased RING1B enrichment (Fig 1A). We also confirmed that H3K9me2 (catalyzed by G9A and GLP) in the TSSs of the late PGC markers decreased upon *Max* KO, whereas H3K9me2 in the TSS of *Hbb-b1* was not affected (S2A Fig), and some of the late PGC markers were up-regulated in *G9a* or *GLP*-KO ESCs (S2B Fig). These results were in agreement with our previous report [2,13] and indicate that MAX is required for the recruitment of PCGF6-PRC1 to its target genes.

To further assess contributions of PCGF6-PRC1 to the regulation of germ cell–related genes under the control of MAX (S3 Table), we compared expression change of these genes in *Max*-KD ESCs [2] and *L3mbtl2*-KD ESCs. *L3mbtl2* disruption has been reported to abolish PCGF-PRC1-mediated gene repression [12]. We found limited up-regulation of germ cell–related genes in *L3mbtl2*-KD ESCs compared to *Max*-KD ESCs (Fig 1B and 1C). Limited up-regulation of the late PGC markers in *Ring1a/b*-DKO ESCs compared with *Max*-null ESCs was also confirmed by qRT-PCR (Figs 1D and S1B), as reported in the previous study [13]. The results suggest that MAX fully repress the expression of germ cell-related genes by PCGF6-PRC1-dependent and independent mechanisms. We also performed Gene Ontology (GO) analysis of up-regulated genes in *Max*-KD ESCs and several ESCs with functional deficiency of the PCGF6-PRC1 complex, including *Ring1a/b*-DKO ESCs [16], *G9a*-KO ESCs [17], *L3mbtl2*-KD ESCs, and *Pcgf6*-KO ESCs [13]. We found germ cell-related genes and/or germ-cell related GO terms in genes up-regulated only in *Max*-KD ESCs as well as in genes commonly upregulated in *Max*-KD ESCs and in those with KD or KO of other factors containing PCGF6-PRC1 (S2C–S2F Fig). The results suggest that PCGF6-PRC1 repress only a subset of germ cell-related genes under the control of MAX.

PCGF6-PRC1 also contains the histone deacetylases HDAC1 and HDAC2 (HDAC1/2) [9], and we confirmed interaction between MAX and HDAC1 using a co-immunoprecipitation assay (S3A and S3B Fig). However, the late PGC markers were not up-regulated, and only a few germ cell–related genes were up-regulated in *Hdac1/2*-DKD ESCs (S3C–S3E Fig), suggesting that contribution of HDAC1/2 on repression of germ cell-related genes in PCGF6-PRC1 is limited. Collectively, these results suggest that MAX-containing PCGF6-PRC1 partially represses subset of germ cell-related genes, but additional MAX-interacting proteins could play a role in further repression of these genes.

### MAX-mediated repression of germ cell–related genes through DNA methylation

**Fig 2:**
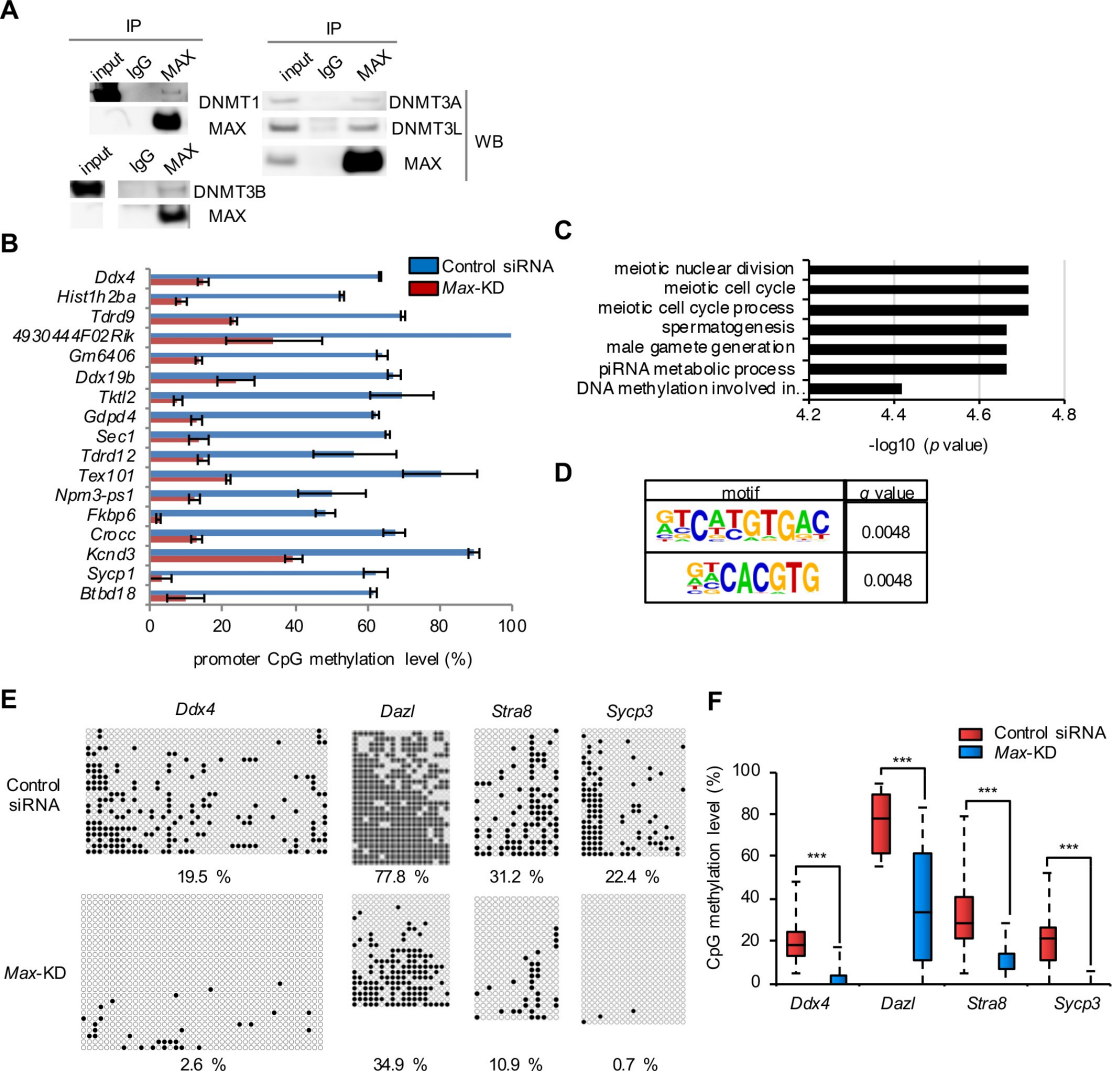
MAX-mediated repression of germ cell–related genes through DNA methylation. (A) Samples immunoprecipitated using anti-MAX antibody or control IgG were analyzed by Western blotting using anti-DNMT antibodies. Principally, the same result was obtained in two independent experiments. The un-cropped data of these images are shown in S4A Fig. (B) Levels of CpG methylation of genes with a DMR in close proximity (±300 bp) for *Max*-KD ESCs and control ESCs from TMS. The ratios of methylated CpGs in the regions ±300 bp of the genes are shown. Values are plotted as mean ± SEM of 2 biological replicates. (C) GO analysis of 17 genes with DMRs hypomethylated in *Max*-KD ESCs compared with control ESCs. GO terms with corrected *P* value < 0.05 (top 7) are shown. (D) Motif analyses of 17 DMRs showed significant enrichment of E-box–like sequences. Motif sequences with the lowest *q* value (top 2) are shown. (E) DNA methylation status of the promoter regions of the late PGC markers in control and *Max*-KD ESCs, as determined by bisulfite sequencing. The filled and open circles indicate methylated- and un-methylated CpGs, respectively. The data shown were combined from two independent experiments. The percentage of methylated CpGs is indicated. (F) Box-whisker plots of the CpG methylation levels shown in Fig 2E. The lines inside the boxes show the median. The whiskers indicate the minimum and maximum. ***P < 0.001 (Mann-Whitney *U*-test).

Since DNMT3B and DNMT1 are involved in repression of germ cell–related genes in PGCs [27,34,35] and we recently identified DNMT3A and DNMT3L as MAX-interacting proteins in ESCs by mass spectrometry (data not shown), we first focused on DNMTs as additional candidate co-repressors associated with MAX. We confirmed co-immunoprecipitation of DNMT1, DNMT3A, DNMT3B, and DNMT3L with MAX in ESCs (Figs 2A and S4A). To elucidate the significance of functional interactions between MAX and DNMTs on a genome-wide level, we performed targeted methylome sequencing (TMS) [19,36] for semi-comprehensive DNA methylome analysis of *Max*-KD ESCs. For consistency with the transcriptome data in the previous study [2], we performed methylome analysis using VV3 ESCs with or without *Max*-KD in this study. A differentially methylated region (DMR) calling identified 17 genes which had hypomethylated DMRs in close proximity (±300 bp) compared with control ESCs (Fig 2B and S4 Table). Notably, germ cell–related GO terms were enriched in these 17 genes (Fig 2C), and motif analyses revealed that the DMRs in *Max*-KD ESCs frequently contain E-box sequences (CACGTG), a binding motif of MAX (Fig 2D). Bisulfite sequence analysis by conventional Sanger sequencing of the late PGC markers in *Max*-KD ESCs confirmed that the levels of TSS DNA methylation were clearly lower than in control ESCs (Fig 2E and 2F). These results demonstrate that MAX associates with DNMTs and contributes to the maintenance of DNA methylation and/or *de novo* methylation of germ cell–related genes.

To verify the contribution of DNA methylation to the repression of germ cell–related genes, existing microarray data for *Max*-KD ESCs and *Dnmts*-TKO ESCs [15,25] were re-analyzed. We found that 266 genes, including *Dazl* and *Stra8*, were commonly up-regulated by *Max*-KD and *Dnmts*-TKO, in which germ cell–related GO terms, especially those involved in meiosis, were enriched (S4B Fig). In addition, 1,245 genes, including *Vasa* and *Sycp3*, were up-regulated by *Max*-KD alone, in which germ cell–related GO terms were also enriched (S4B Fig). By contrast, germ cell–related GO terms were not enriched in genes up-regulated by *Dnmts*-TKO alone. Consistent with these results, we confirmed up-regulation of *Dazl* and *Stra8*, but not *Ddx4* and *Sycp3*, in *Dnmts*-TKO ESCs by qRT-PCR (S4C Fig), and levels of upregulation of *Dazl* and *Stra8* by *Dnmts*-TKO was similar as those in *Max*-null ESCs (S1B Fig and S5 Table). These results indicate that MAX represses some germ cell–related genes through DNA methylation.

### MAX-mediated repression of germ cell–related genes through H3K9me3

Since *Sycp3* was not significantly up-regulated in any ES cell lines examined except *Max*-KD or *Max*-null ESCs (Figs 1C, 1D and S4C) [2], *Sycp3* may be repressed by a mechanism that does not dependent on PCGF6-PRC1 nor DNMTs, but dependent on MAX. Thus, we hypothesized that additional factors associate with MAX to cooperatively repress germ cell–related genes, including *Sycp3*. Using previously reported ChIP-seq data for ESCs, we found that SETDB1 (a histone methyltransferase that catalyzes H3K9me3) localized in the TSSs of the late PGC markers, including *Sycp3* (S5A Fig) [37]. Data in a previous study also showed that some germ cell-related genes were targets of SETDB1 and H3K9me3 in ESCs [38].

**Fig 3:**
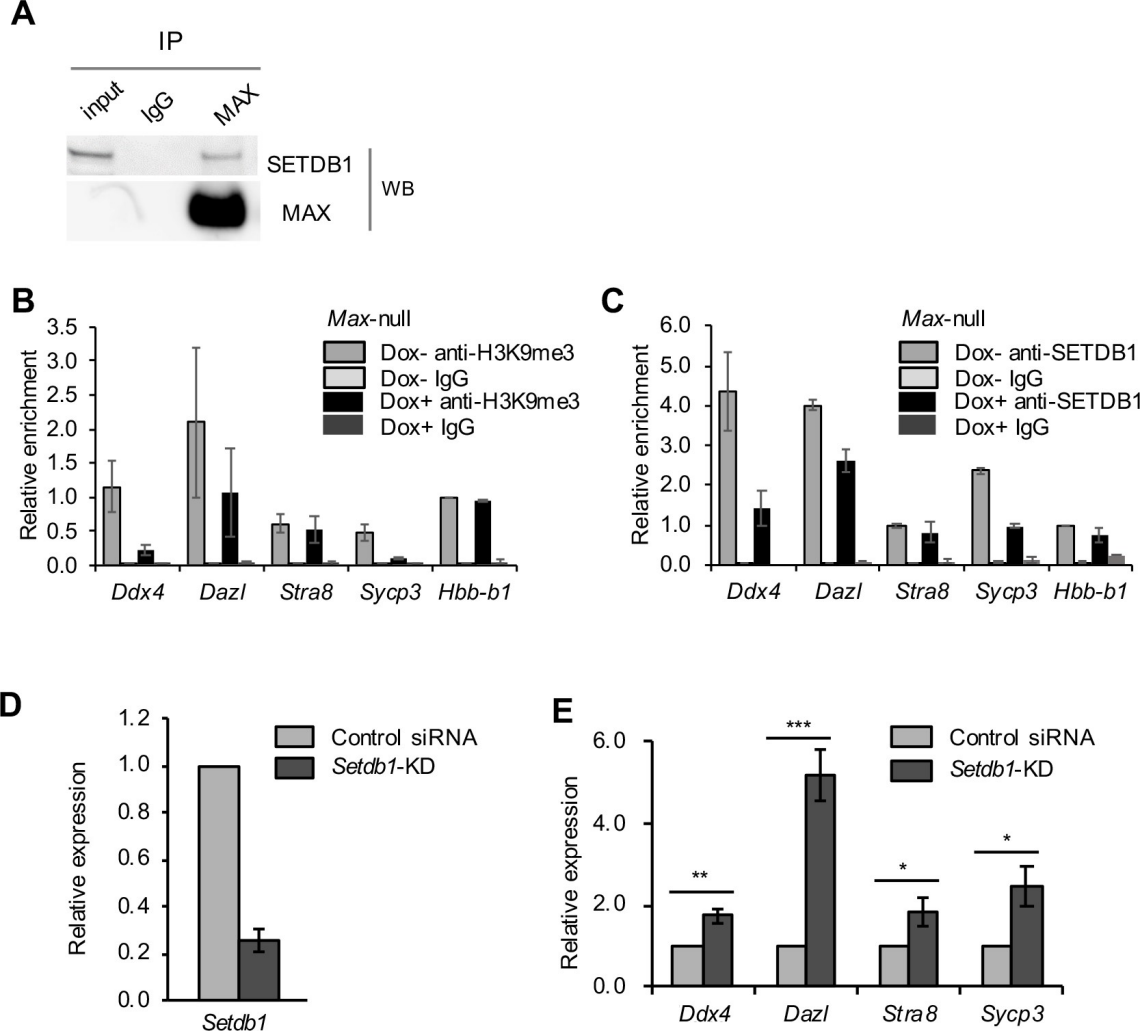
MAX-mediated repression of germ cell–related genes through H3K9me3. (A) Samples immunoprecipitated using anti-MAX antibody or control IgG were analyzed by Western blotting using anti-SETDB1 antibody. Principally, the same result was obtained in two independent experiments. The un-cropped data of this image is shown in S5B Fig. (B, C) ChIP-qPCR analyses of *Max*-null ESCs (Dox+) and control ESCs (Dox−) using anti-H3K9me3 antibody (B), anti-SETDB1 antibody (C) or control IgG. The data are displayed in the same way as in Fig 1A. (D) KD efficiency of *Setdb1* in ESCs at day 2 post-siRNA treatment, as determined by RT-qPCR. (E) Relative expression of the late PGC markers in *Setdb1*-KD ESCs, as determined by qRT-PCR. The expression in control ESCs was set as 1.0. Values are plotted as the mean ± SEM of 3 biological replicates. *P < 0.05, **P < 0.01, ***P < 0.001 (Student’s *t*-test).

To test the possible cooperative role of MAX and SETDB1 in repressing germ cell–related genes, we first examined the interaction between MAX and SETDB1 using a co-immunoprecipitation assay (Figs 3A and S5B). We then examined MAX dependency of SETDB1 localization in the late PGC markers. The levels of H3K9me3 and SETDB1 in the TSSs of the late PGC markers (except *Stra8*) were decreased in *Max*-null ESCs compared with control ESCs, whereas levels of H3K9me3 and SETDB1 in the TSS of *Hbb-b1* did not significantly differ from controls (Fig 3B and 3C).

To verify whether the decline in H3K9me3 levels causes up-regulation of germ cell–related genes, previously reported microarray data for *Setdb1*-KO ESCs [26] were re-analyzed. We found that 238 genes, including *Vasa*, *Dazl*, and *Sycp3*, were commonly up-regulated by *Max*-KD and *Setdb1*-KO, and germ cell–related GO terms were enriched in these genes (S5C Fig). In addition, 1,274 genes, including *Stra8*, were up-regulated by *Max*-KD alone, and germ cell–related GO terms were also enriched in these genes. By contrast, germ cell–related GO terms were not enriched in genes up-regulated by *Setdb1*-KO alone. The results suggest that MAX cooperatively represses a subset of germ cell-related genes with SETDB1. Furthermore, *Setdb1*-KD ESCs showed higher expression of all of the late PGC markers including *Sycp3* than control ESCs as in *Max*-null ESCs (S1B Fig), and *Dazl* was particularly up-regulated compared with other genes (Fig 3D and 3E). The results together suggest that MAX-dependent recruitment of SETDB1 catalyzes H3K9me3 in a subset of germ cell–related genes, resulting in their repression.

### Relationship between DNA methylation, H3K9me3, and PCGF6-PRC1 in repression of germ cell–related genes

**Fig 4:**
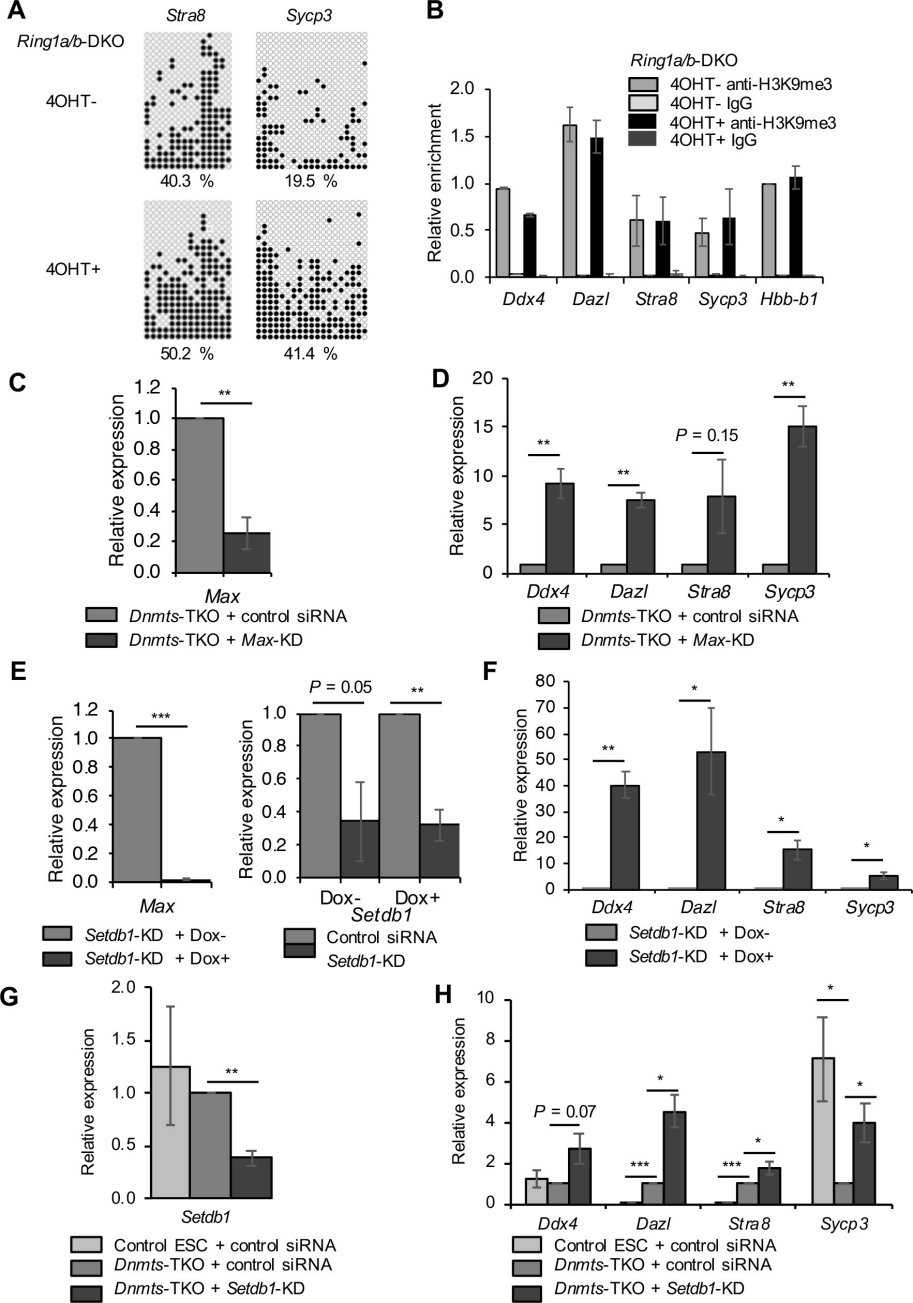
RING1A/B-independent DNA and H3K9 methylation, and a cooperative effect of *Dnmts*-TKO and *Setdb1*-KD on repression of the late PGC markers. (A) DNA methylation status of the promoter regions of *Stra8* and *Sycp3* in control (4OHT−) and *Ring1a/b*-DKO ESCs (4OHT+) determined by bisulfite sequencing. The data are displayed in the same way as in Fig 2E. (B) ChIP-qPCR analyses of *Ring1a/b*-DKO ESCs (4OHT+) and control ESCs (4OHT−) using anti-H3K9me3 antibody or control IgG. The data are displayed in the same way as in Fig 1A. (C) KD efficiency of *Max* in *Dnmts*-TKO ESCs at day 3 post-siRNA treatment, as determined by RT-qPCR. (D) Relative expression of the late PGC markers in *Max*-KD/*Dnmts*-TKO ESCs, as determined by qRT-PCR. The expression in *Dnmts*-TKO ESCs with control siRNA treatment was set as 1.0. (E) KO and KD efficiency of *Max* (left panel) and *Setdb1* (right panel) in *Setdb1*-KD/*Max*-null ESCs at day 3 post-siRNA treatment, as determined by RT-qPCR. KD efficiency of *Setdb1* is normalized with the expression of *Setdb1* in *Max*-null ESCs with control siRNA treatment. (F) Relative expression of the late PGC markers in *Setdb1*-KD/*Max*-KO ESCs, as determined by qRT-PCR. The expression in *Setdb1*-KD ESCs with *Max* expression (Dox-) was set as 1.0. (G) KD efficiency of *Setdb1* in *Dnmts*-TKO ESCs at day 2 post-siRNA treatment, as determined by RT-qPCR. (H) Relative expression of the late PGC markers in *Setdb1*-KD/*Dnmts*-TKO ESCs, as determined by qRT-PCR. The expression in *Dnmts*-TKO ESCs with control siRNA treatment was set as 1.0. Values are plotted as the mean ± SEM of 3 biological replicates. *P < 0.05, **P < 0.01, ***P < 0.001 (Student’s *t*-test).

Although DNMTs and SETDB1 have not been identified as components of PCGF6-PRC1 [12,13,39], we investigated whether or not DNA methylation and H3K9me3 are regulated by PCGF6-PRC1. To this end, we determined the levels of DNA methylation and H3K9me3 in the TSSs of the late PGC markers in *Ring1a/b*-DKO ESCs. Bisulfite sequence analyses revealed that *Ring1a/b*-DKO did not decrease the level of DNA methylation in the *Stra8* and *Sycp3* TSSs (Fig 4A) or the level of H3K9me3 in the TSSs of the late PGC markers (Fig 4B). These data suggest that the regulation of DNA methylation and H3K9me3 on the late PGC markers are independent of PCGF6-PRC1.

We also investigated to what extent DNMTs and SETDB1 contribute to the repression of germ cell-related genes through MAX-mediated pathways. *Max*-KD in *Dnmts*-TKO ESCs (Fig 4C and 4D), as well as *Max*-KO in *Setdb1*-KD ESCs (Fig 4E and 4F), remarkably enhanced the expression of the late PGC markers. These results suggest that DNMTs and SETDB1 are partially contribute for the repression of germ cell–related genes mediated by MAX, and complete repression of these genes are achieved through multiple epigenetic function based on MAX.

We further investigated the relationship between DNA methylation and H3K9me3. *Setdb1* KD in *Dnmts*-TKO ESCs caused additional up-regulation of the late PGC markers, especially *Dazl* and *Stra8*, compared with *Dnmts*-TKO ESCs exposed to control siRNA (Fig 4G and 4H). The expression of *Sycp3* was clearly decreased in *Dnmts*-TKO ESCs compared with control ESCs, indicating that DNA methylation resulted in transcriptional activation for *Sycp3* as is the case with a previous report [40]. These results suggest that DNMTs and SETDB1 function through distinct pathways for the repression of germ cell–related genes. Taken together with GO analysis of up-regulated genes in *Max*-KD ESCs and in those with functional deficiency of PCGF6-PRC1-containing epigenetic factors (S2C–S2F, S4B and S5C Figs), several types of PCGF6-PRC1-indenendent machineries would have different target genes for germ cell-related gene repression.

### Fractionation of MAX-interacting complexes

**Fig 5:**
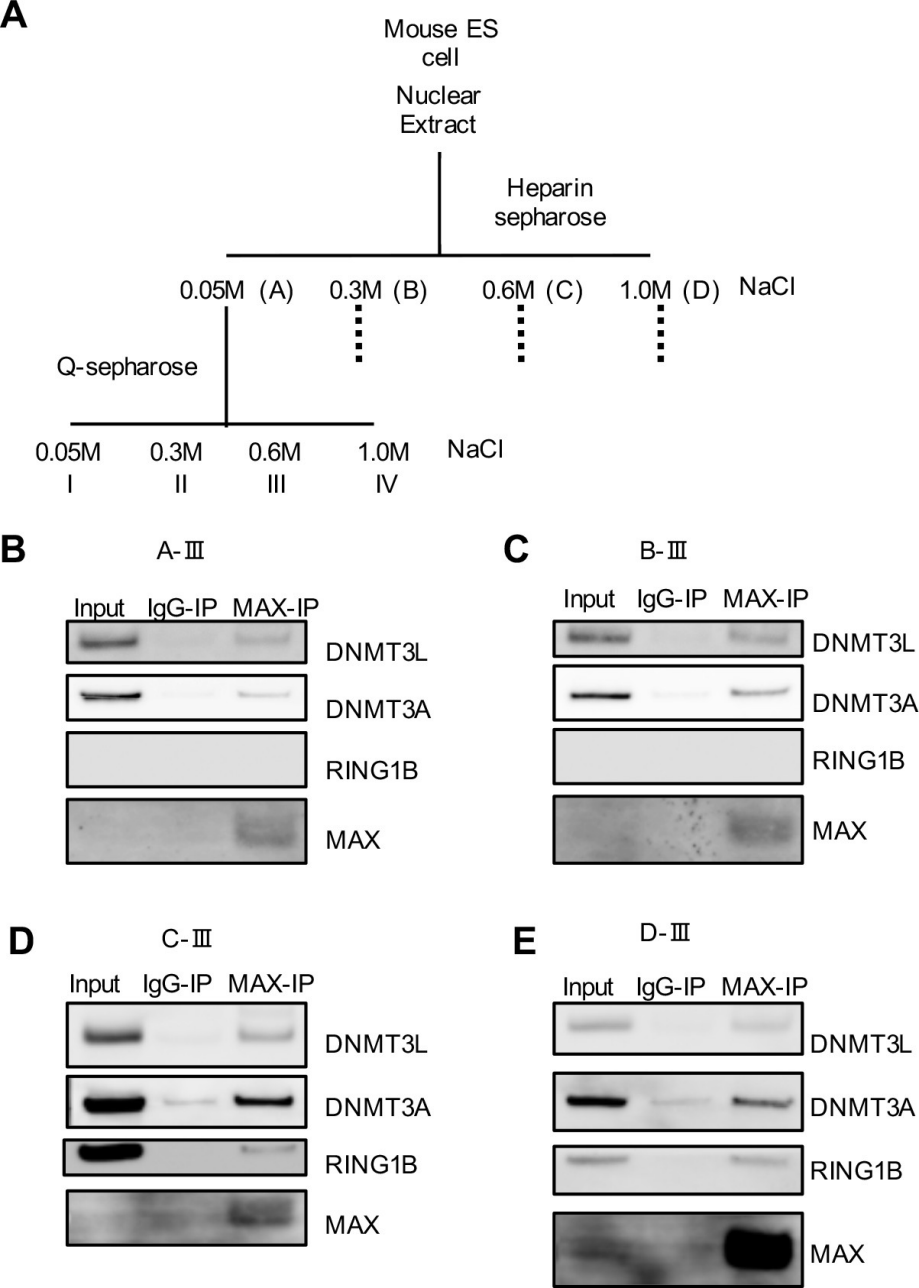
Fractionation of MAX-interacting complexes. (A) Schematic illustration of the fractionation of ESC nuclear extracts by column chromatography. Nuclear extracts were prepared from VV3 ESCs and fractionated into fractions A-D on a heparin sepharose column. Fractions A-D were further fractionated into fractions I-IV on a Q sepharose column. (B to E) Samples immunoprecipitated using anti-MAX antibody or control IgG for fraction A-III (B), B-III (C), C-III (D), or D-III (E) were analyzed by Western blotting using anti-DNMT, anti-RING1B, or anti-MAX antibodies. Principally, the same result was obtained in two independent experiments. The un-cropped data of these images are shown in S6A–S6D Fig, respectively.

The abovementioned results suggest that MAX functions in the repression of germ cell–related genes by forming multiple complexes with various epigenetic factors in addition to PCGF6-PRC1. To further clarify this possibility, we sequentially fractionated ESC nuclear extracts using heparin sepharose and Q sepharose columns. Each fraction collected was subjected to immunoprecipitation using an anti-MAX antibody with subsequent Western blotting (Fig 5A). We observed co-immunoprecipitation of MAX, DNMT3A and DNMT3L with or without RING1B in some fractions (Figs 5B–5E and S6A–S6D), suggesting the existence of both PCGF6-PRC1-associated and–unassociated MAX-DNMT complexes. In D-III fraction, the MAX signal was strongest but the signals of RING1B, DNMT3A and DNMT3L were comparable to other fractions. It suggests that D-III fraction contains multiple MAX-mediated complexes including PCGF6-PRC1. These results suggest that MAX, DNMT3A, and/or DNMT3L could regulate DNA methylation independent from PCGF6-PRC1 in ESCs.

## Discussion

In this study, we showed that MAX represses germ cell–related genes in ESCs via interaction with the epigenetic regulators DNMTs and SETDB1 in addition to PCGF6-PRC1. The target genes of MAX/L3MBTL2-containing PCGF6-PRC1, MAX-DNMT1, and MAX-SETDB1 in ESCs may partially overlap, which suggests that those complexes redundantly repress some of their target genes, but many genes upregulated by *Max*-KD were affected either by *L3mbtl2*-KD, *G9a*-KO, *Dnmts*-TKO, or *Setdb1*-KO alone (S7A Fig). As PRCs are required for the establishment of H3K27me3 and subsequent silencing of developmental genes in bivalent domains involving both a repressive modification (H3K27me3) and a permissive modification (H3K4me3) [41], we examined the overlap between bivalent genes and genes regulated by MAX (S7B Fig). A majority of bivalent genes in ESCs [42,43] did not overlap with genes up-regulated by *Max*-KD. We further revealed the presence of complexes composed of MAX and DNMTs without RING1B in ESCs (Fig 5B and 5C). Collectively, these data suggest that MAX is involved in multiple regulatory mechanisms that differ from that involving PRC1, according to the target genes. Furthermore, MAX-DNMTs and MAX-SETDB1 may function, at least to some degree, as part of distinct pathways to repress their target genes, because *Dnmts*-TKO and *Setdb1*-KD additively repressed the expression of the late germ cell markers (Fig 4G and 4H). It was also reported that *Dnmts*-TKO ESCs exhibit minimal changes in genome-wide H3K9me3 occupancy compared with wild-type ESCs, supporting the hypothesis that DNA methylation and H3K9me3 act non-redundantly in ESCs [44]. An important future task would be to identify MAX-containing complexes other than PCGF6-PRC1 that repress germ cell–related genes.

We summarized the quantitative data for RT-qPCR (fold changes), ChIP-qPCR (fold changes of localization), and bisulfite sequence (Δ %CpGme) compared with each control condition obtained in this study (S5 Table). *Ddx4* showed notable decrease of H3K9me3 (0.196 fold), SETDB1 localization (0.329 fold) and DNA methylation (- 16.9%) by knockout or knockdown of *Max*, but showed only a subtle up-regulation by *Setdb1*-KD (1.89 fold) and no expression change by *Dnmts*-TKO (1.19 fold). Since *Ddx4* was highly up-regulated by *Ring1a/b*-DKO (5.25 fold) or *G9a*-KO (3.65 fold), both of which are components of PCGF6-PRC1, *Ddx4* may be mainly repressed by PCGF6-PRC1 and have resistance to the perturbation of H3K9me3 and DNA methylation.

On the other hand, *Ring1a/b*-DKO had moderate or no effects for the expression of *Dazl* (2.06 fold), *Stra8* (2.06 fold) and *Sycp3* (0.77 fold) compared with the effect for *Ddx4*, indicating the existence of additional machineries for their repression. Meanwhile, *Dazl* and *Stra8* were remarkably up-regulated by *Dnmts*-TKO (*Dazl*: 27.11 fold, *Stra8*: 12.60 fold) and their DNA methylation was decreased (*Dazl*: - 42.9%, *Stra8*: - 20.3%) by *Max*-KD. They were also up-regulated by *Setdb1*-KD (*Dazl*: 5.18 fold, *Stra8*: 1.86 fold) and their H3K9me3 (*Dazl*: 0.507 fold, *Stra8*: 0.843 fold) and localization of SETDB1 (*Dazl*: 0.650 fold, *Stra8*: 0.805 fold) were decreased by *Max*-KO. These changes were larger in *Dazl* than in *Stra8*. The results together suggest that DNA methylation and H3K9me3 make a major contribution on repression of *Dazl* and *Stra8*, and *Dazl* is more strongly controlled by DNMTs and SETDB1 than *Stra8*. *Dazl* and *Stra8* were also up-regulated by *GLP*-KO (*Dazl*: 7.10 fold, *Stra8*: 3.29 fold) and by *G9a*-KO (*Dazl*: 5.17 fold). Taken together, these data indicate regulation for *Dazl* and *Stra8* by various chromatin-modifying complexes including PCGF6-PRC1. In addition, the expression level of *Dazl* (119 fold) and *Stra8* (80 fold) were additively increased in *Dnmts*-TKO ESCs with *Setdb1*-KD compared to control ESCs with control siRNA (Fig 4H and S5 Table). Since these fold changes were higher than those in *Max*-null ESCs (*Dazl*: 32 fold, *Stra8*: 13 fold) (S1B Fig and S5 Table), DNMTs and /or SETDB1 may have additional roles for the repression of *Dazl* and *Stra8* other than MAX-containing complexes.

*Sycp3* showed decrease of H3K9me3 (0.205 fold) and SETDB1 localization (0.407 fold) by *Max*-KO and its expression was up-regulated by *Setdb1*-KD (2.48 fold). Though *Sycp3* also showed considerable decrease of DNA methylation (- 21.7%) by *Max*-KD (Fig 2E and 2F), *Dnmts*-TKO rather down-regulated *Sycp3* expression (0.26 fold, Fig 4H). The results suggest that DNMTs and DNA methylation do not result in transcriptional repression on *Sycp3*, and SETDB1 and H3K9me3 make a major contribution on its repression. In addition, *Sycp3* was not up-regulated by *Ring1a/b*-DKO (0.77 fold) and by *GLP-KO* (0.87 fold), and therefore PCGF6-PRC1 may not play a role on repression of *Sycp3*. Although these four genes are all categorized as the late PGC markers, the regulatory mechanisms and responsible modifications seem quite different one another.

Although *Dnmts*-TKO ESCs exhibited robust growth and maintained their undifferentiated characteristics [15], DNMTs reportedly play several important roles in ESCs. DNMT1 and DNMT3A/3B suppress long terminal repeats and long interspersed elements of retrotransposons, respectively, possibly through interaction with UHRF1 [45]. Furthermore, DNA methylation in imprinting control regions (ICRs) by DNMT3A/3B is stably maintained via the interaction between G9a and GLP [46]. In this study, we demonstrated interactions between DNMTs and MAX, and preferential enrichment of DMRs at the promoters of meiotic genes in ESCs after *Max*-KD (Fig 2C), suggesting additional roles for DNMTs in the repression of meiotic programming in association with MAX as discussed below.

SETDB1 also plays a variety of roles in ESCs. Several studies have reported that SETDB1 and ATF7IP form a complex and that both are required for proviral silencing in mouse ESCs, especially for class I and II endogenous retroviruses (ERVs) [44,47,48]. We examined whether MAX is also involved in the regulation of ERVs silenced by SETDB1 and ATF7IP. The expression of MLV and IAP Ez (class I and II ERVs, respectively) and a class III ERV MaLR was not affected by *Max*-KD, whereas the expression of another class III ERV, MERVL, was significantly higher in *Max*-KD ESCs than in control ESCs (S8 Fig). These results suggest that MAX does not function in concert with the ATF7IP-SETDB1 complex with regard to proviral silencing. It has been reported that a transcription regulator, KAP1/TRIM28, is enriched in MaLR and MERVL at a similar level, but only MERVL is upregulated in *Kap1*-KO ESCs [49]. It suggests that KAP1/TRIM28 represses MERVL, but not MaLR in class III ERV. Since we have found the interaction between MAX and KAP1 in ESCs by the immunoprecipitation of FLAG-tagged MAX protein (unpublished data), MAX may exert silencing particular ERVs including MERVL with KAP1, but not with SETDB1.

As described above, MAX is associated with several epigenetic mechanisms that lead to repression of germ cell–related genes in ESCs, but whether the mechanisms in ESCs are also functional in PGCs is unclear. DNMT1 preserves DNA methylation of ICRs and meiotic gene promoters in PGCs, and conditional deletion of *Dnmt1* in PGCs was shown to cause a decrease in the number of germ cells and their precocious differentiation, including up-regulation of spermatogenesis- and meiosis-related genes in male and female germ cells, respectively, consequently leading to hypogonadism and infertility [27]. *Setdb1* KO E13.5 PGCs exhibit de-repression of many ERVs, a reduced number of male PGCs, and postnatal hypogonadism [28]. We re-analyzed existing data to estimate whether germ cell–related genes repressed by MAX in ESCs are also repressed by DNMTs or SETDB1 in PGCs. Eighty-five genes, defined as germ cell–related according to the GO term “reproduction”, were found to be up-regulated by *Max*-KD in ESCs (S3 Table). Among those, up-regulated genes in *Dnmt1*-cKO or *Setdb1*-cKO PGCs were extracted as genes with > 1.3-fold change compared with control PGCs. 52 and 55 genes were found to be up-regulated by *Dnmt1*-cKO and *Setdb1*-cKO in female E13.5 PGCs, respectively, whereas 32 and 41 genes were found to be up-regulated by *Dnmt1*-cKO and *Setdb1*-cKO in male E13.5 PGCs (S9 Fig). Considering that decreases in MAX levels induce meiosis-like cytologic changes in cultured germline stem cells [3], MAX, DNMTs, and SETDB1 could repress meiosis-related genes in PGCs in a similar manner as in ESCs. Future research should focus on determining whether MAX, DNMTs, and/or SETDB1 function in concert to control initiation of meiosis in PGCs.

## Supporting information

S1 Fig Repression of the late PGC markers by *Max* KO in ESCs. (A) Dox-dependent attenuation of MAX protein levels in *Max*-null ESCs was assessed by Western blotting using anti-MAX antibody. Principally, the same result was obtained in two independent experiments. (B) Relative expression of the late PGC markers in *Max*-null ESCs (Dox+), as determined by qRT-PCR. The expression in control ESCs (Dox−) was set as 1.0. Values are plotted as the mean ± SEM of 3 biological replicates. **P < 0.01, ***P < 0.001 (Student’s *t*-test). (C) ChIP-qPCR analyses of *Max*-null ESCs (Dox+) and control ESCs (Dox−) using anti-MAX antibody or control IgG for the promoter region of the late PGC markers and hemoglobin β (*Hbb-b1*) as a negative control of MAX localization. The data are displayed in the same way as in Fig 1A.
(TIF)

S2 Fig Partial contribution of PCGF6-PRC1 to Max-mediated gene repression. (A) ChIP-qPCR analyses of *Max*-null ESCs (Dox+) and control ESCs (Dox−) using anti-H3K9me2 antibody or control IgG. The data are displayed in the same way as in Fig 1A. (B) Relative expression of the late PGC markers in *G9a-* or *GLP*-KO ESCs as determined by qRT-PCR. The expression in control ESCs (TT2) was set as 1.0. Values are plotted as the mean ± SEM of 3 biological replicates. *P < 0.05, **P < 0.01, ***P < 0.001 (Student’s *t*-test). (C to F) Venn diagram of genes up-regulated in *Max*-KD ESCs (GSE45181) [2] (*n* = 3, > 2-fold change, one-way ANOVA *P* < 0.05) compared with genes up-regulated in *L3mbtl2*-KD ESCs (*n* = 4, > 2-fold change, one-way ANOVA *P* < 0.05) (C), *G9a*-KO ESCs (*n* = 1, > 1.3-fold change) (D), *Ring1a/b*-DKO ESCs (GSE10573) [16] (*n* = 1, > 1.5-fold change) (E), or *Pcgf6*-KO ESCs (GSE84480) [13] (*n* = 2, > 2-fold change) (F). GO analyses of genes representing each category were performed. GO terms with the lowest corrected *P* value (top 7) are shown.
(TIF)

S3 Fig HDAC1/2 represses some germ cell–related genes in ESCs. (A) Immunoprecipitated samples using anti-MAX antibody or control IgG were analyzed by Western blotting using anti-HDAC1 antibody. Principally, the same result was obtained in two independent experiments. (B) Un-cropped data of Western blotting corresponding to S3A Fig. Immunoprecipitated samples by anti-MAX antibody or control IgG were subjected to Western blotting by using anti-HDAC1 antibody. Red indicates the data shown in S3A Fig. (C) KD efficiency of *Hdac1* and *Hdac2* in ESCs at day 2 post-siRNA treatment. (D) Relative expression of germ cell-related genes (*Rhox10*, *Sohlh2*, *Tex101*, *Tex19*.*1*, and *Tex19*.*2*) in *Max*-KD ESCs at day 3 post-siRNA treatment (4 biological replicates), as determined by qRT-PCR. (E) Relative expression of the late PGC marker genes, *Rhox10*, *Sohlh2*, *Tex101*, *Tex19*.*1*, and *Tex19*.*2* in *Hdac1/2*-DKD ESCs at day 3 post-siRNA treatment (3 biological replicates), as determined by qRT-PCR. The expression in control ESCs was set as 1.0. Values are plotted as the mean ± SEM. n.s; not significant, *P < 0.05, **P < 0.01, ***P < 0.001 (Student’s *t*-test).
(TIF)

S4 Fig DNMTs contribute to the repression of germ cell–related genes. (A) Un-cropped data of Western blotting corresponding to Fig 2A. Immunoprecipitated samples by anti-MAX antibody or control IgG were subjected to Western blotting by using anti-DNMT antibodies. Red indicates the data shown in Fig 2A. The elution was performed twice and each eluted sample was analyzed separately. (B) Venn diagram of genes up-regulated in *Max*-KD ESCs (GSE45181) [2] and *Dnmts*-TKO ESCs (GSE20177) [25] (*Max*-KD ESCs; *n* = 3, > 2-fold change, one-way ANOVA *P* < 0.05, *Dnmts*-TKO ESCs; *n* = 2, > 1.3-fold change). GO analyses of genes representing each category were performed. GO terms with the lowest corrected *P* value (top 7) are shown. (C) Relative expression of the late PGC markers in *Dnmts*-TKO ESCs determined by qRT-PCR. The expression in control ESCs was set as 1.0. Values are plotted as the mean ± SEM of 3 biological replicates. *P < 0.05, ***P < 0.001 (Student’s *t*-test).
(TIF)

S5 Fig Relationship between Max and Setdb1 in repression of germ cell–related genes in ESCs. (A) ChIP-seq data for SETDB1 in ESCs (GSE73434) [37] were re-analyzed using Integrative Genomics Viewer (IGV). Neighboring regions of TSSs of the late PGC markers are shown. (B) Un-cropped data of Western blotting corresponding to Fig 3A. Immunoprecipitated samples by anti-MAX antibody or control IgG were subjected to Western blotting by using anti-SETDB1 antibody. Red indicates the data shown in Fig 3A. (C) Venn diagram of genes up-regulated in *Max*-KD ESCs (GSE45181) [2] and *Setdb1*-KO ESCs (GSE28593) [26] (*Max*-KD ESCs; *n* = 3, > 2-fold change, one-way ANOVA *P* < 0.05, *Setdb1*-KO ESCs; *n* = 3, > 1.3-fold change, one-way ANOVA *P* < 0.05). GO analyses of genes representing each category were performed. GO terms with the lowest corrected *P* value (top 7) are shown.
(TIF)

S6 Fig Fractionation of MAX-interacting complexes (un-cropped data). (A to D) Un-cropped data of Western blotting corresponding to Fig 5B–5E, respectively. Immunoprecipitated samples by anti-MAX antibody or control IgG were subjected to Western blotting by using anti-DNMT3A, DNMT3L, RING1B antibodies for fraction A-III (A), B-III (B), C-III (C), or D-III (D). Red indicates the data shown in Fig 5B–5E, respectively.
(TIF)

S7 Fig Relationships between MAX, L3MBTL2, G9A, DNMTs, and SETDB1 in repression of germ cell–related genes in ESCs. (A) Venn diagram of genes up-regulated in *L3mbtl2*-KD ESCs (*n* = 4, > 2-fold change, one-way ANOVA *P* < 0.05), *G9a*-KO ESCs (*n* = 1, > 1.3-fold change), *Setdb1*-KO ESCs (GSE28593) [26] (*n* = 3, > 1.3-fold change, one-way ANOVA *P* < 0.05), and *Dnmts*-TKO ESCs (GSE20177) [25] (*n* = 2, > 1.3-fold change) among up-regulated genes in *Max*-KD ESCs (GSE45181) [2] (*n* = 3, > 2-fold change, one-way ANOVA *P* < 0.05). (B) Venn diagram showing relationships between genes up-regulated in *Max*-KD ESCs [2] and the bivalent genes [42,43].
(TIF)

S8 Fig Regulation of ERVs via MAX. Relative expression of class I-III ERVs in *Max*-KD ESCs, as determined by qRT-PCR. The expression in VV3 ESCs treated with control siRNA was set as 1.0. Values are plotted as the mean ± SEM of 3 biological replicates. n.s.: not significant, ***P < 0.001 (Student’s *t*-test).
(TIF)

S9 Fig Expression change of germ cell-related genes in *Dnmt1*^cKO^ and *Setdb1*^cKO^ PGCs. (A and B) Germ cell–related genes up-regulated in *Max*-KD ESCs compared with control ESCs (85 genes, *n* = 3, > 2-fold change, ANOVA *P* < 0.05, with GO term “reproduction”, S3 Table) were selected and expression change of these genes in E13.5 *Setdb1*^cKO^ PGCs (GSE60377) [28] (*n* = 2) (A) or *Dnmt1*^cKO^ PGCs (GSE74938) [27] (*n* = 3) (B) compared with control PGCs were represented as heat maps.
(TIF)

S1 Table List of primers used in this study. (TIF)

S2 Table List of antibodies used in this study. (TIF)

S3 Table List of germ cell-related genes up-regulated in *Max*-KD ESCs compared with control ESCs. Eighty-five of germ cell–related genes with gene ontology term “reproduction” were extracted as up-regulated genes in *Max*-KD ESCs compared with control ESCs (GSE45181) [2] (*n* = 3, > 2-fold change, one-way analysis of variance [ANOVA] *P* < 0.05).
(TIF)

S4 Table List of genes with differentially methylated region (DMR). (TIF)

S5 Table Summaries of qPCR, ChIP and bisulfite sequence in this study. Red and orange indicate > 5 fold and > 2 fold up-regulated genes in RT-qPCR, respectively.
(TIF)

## References

[1] Orkin SH, Hochedlinger K. Chromatin connections to pluripotency and cellular reprogramming. Cell. 2011; 145: 835–850. 10.1016/j.cell.2011.05.019 21663790 PMC4858411

[2] Maeda I, Okamura D, Tokitake Y, Ikeda M, Kawaguchi H, Mise N, et al Max is a repressor of germ cell-related gene expression in mouse embryonic stem cells. Nat Commun. 2013; 4: 1754 10.1038/ncomms2780 23612295

[3] Suzuki A, Hirasaki M, Hishida T, Wu J, Okamura D, Ueda A, et al Loss of MAX results in meiotic entry in mouse embryonic and germline stem cells. Nat Commun. 2016; 7: 11056 10.1038/ncomms11056 27025988 PMC4820925

[4] Hishida T, Nozaki Y, Nakachi Y, Mizuno Y, Okazaki Y, Ema M, et al Indefinite self-renewal of ESCs through Myc/Max transcriptional complex-independent mechanisms. Cell Stem Cell. 2011; 9: 37–49. 10.1016/j.stem.2011.04.020 21726832

[5] Sparmann A, van Lohuizen M. Polycomb silencers control cell fate, development and cancer. Nat Rev Cancer. 2006; 6: 846–856. 10.1038/nrc1991 17060944

[6] Simon JA, Kingston RE. Mechanisms of polycomb gene silencing: knowns and unknowns. Nat Rev Mol Cell Biol. 2009; 10: 697–708. 10.1038/nrm2763 19738629

[7] Luis NM, Morey L, Di Croce L, Benitah SA. Polycomb in stem cells: PRC1 branches out. Cell Stem Cell. 2012; 11: 16–21. 10.1016/j.stem.2012.06.005 22770239

[8] Aloia L, Di Stefano B, Di Croce L. Polycomb complexes in stem cells and embryonic development. Development. 2013;140: 2525–2534. 10.1242/dev.091553 23715546

[9] Gao Z, Zhang J, Bonasio R, Strino F, Sawai A, Parisi F, et al PCGF homologs, CBX proteins, and RYBP define functionally distinct PRC1 family complexes. Mol Cell. 2012; 45: 344–356. 10.1016/j.molcel.2012.01.002 22325352 PMC3293217

[10] Ogawa H, Ishiguro K, Gaubatz S, Livingston DM, Nakatani Y. A complex with chromatin modifiers that occupies E2F- and Myc-responsive genes in G0 cells. Science. 2002; 296: 1132–1136. 10.1126/science.1069861 12004135

[11] Trojer P, Cao AR, Gao Z, Li Y, Zhang J, Xu X, et al L3MBTL2 protein acts in concert with PcG protein-mediated monoubiquitination of H2A to establish a repressive chromatin structure. Mol Cell. 2011; 42: 438–450. 10.1016/j.molcel.2011.04.004 21596310 PMC3142354

[12] Qin J, Whyte WA, Anderssen E, Apostolou E, Chen HH, Akbarian S, et al The polycomb group protein L3mbtl2 assembles an atypical PRC1-family complex that is essential in pluripotent stem cells and early development. Cell Stem Cell. 2012; 11: 319–332. 10.1016/j.stem.2012.06.002 22770845 PMC3647456

[13] Endoh M, Endo TA, Shinga J, Hayashi K, Farcas A, Ma KW, et al PCGF6-PRC1 suppresses premature differentiation of mouse embryonic stem cells by regulating germ cell-related genes. Elife 2017; 6: pii: e21064. 10.7554/eLife.21064 PMC5375644 28304275

[14] Yokobayashi S, Liang CY, Kohler H, Nestorov P, Liu Z, Vidal M, et al PRC1 coordinates timing of sexual differentiation of female primordial germ cells. Nature. 2013; 190: 1954–1955. 10.1038/nature11918 23486062

[15] Tsumura A, Hayakawa T, Kumaki Y, Takebayashi S, Sakaue M, Matsuoka C, et al Maintenance of self-renewal ability of mouse embryonic stem cells in the absence of DNA methyltransferases Dnmt1, Dnmt3a and Dnmt3b. Genes Cells. 2006; 11: 805–814. 10.1111/j.1365-2443.2006.00984.x 16824199

[16] Endoh M, Endo TA, Endoh T, Fujimura Y, Ohara O, Toyoda T, et al Polycomb group proteins Ring1A/B are functionally linked to the core transcriptional regulatory circuitry to maintain ES cell identity. Development. 2008; 135: 1513–1524. 10.1242/dev.014340 18339675

[17] Tachibana M, Sugimoto K, Nozaki M, Ueda J, Ohta T, Ohki M, et al G9a histone methyltransferase plays a dominant role in euchromatic histone H3 lysine 9 methylation and is essential for early embryogenesis. Genes Dev. 2002;16: 1779–1791. 10.1101/gad.989402 12130538 PMC186403

[18] Sekinaka T, Hayashi Y, Noce T, Niwa H, Matsui Y. Selective de-repression of germ cell-specific genes in mouse embryonic fibroblasts in a permissive epigenetic environment. Sci Rep. 2016; 6: 32932 10.1038/srep32932 27608931 PMC5016969

[19] Miura F, Ito T. Highly sensitive targeted methylome sequencing by post-bisulfite adaptor tagging. DNA Res. 2015; 22: 13–18. 10.1093/dnares/dsu034 25324297 PMC4379973

[20] Kobayashi H, Sakurai T, Imai M, Takahashi N, Fukuda A, Obata Y, et al Contribution of intragenic DNA methylation in mouse gametic DNA methylomes to establish oocyte-specific heritable marks. PLoS Genet. 2012; 8: e1002440 10.1371/journal.pgen.1002440 22242016 PMC3252278

[21] Kobayashi H, Sakurai T, Miura F, Imai M, Mochiduki K, Yanagisawa E, et al High-resolution DNA methylome analysis of primordial germ cells identifies gender-specific reprogramming in mice. Genome Res. 2013; 23: 616–627. 10.1101/gr.148023.112 23410886 PMC3613579

[22] Monk D. Germline-derived DNA methylation and early embryo epigenetic reprogramming: the selected survival of imprints. Int J Biochem Cell Biol. 2015; 67: 128–138. 10.1016/j.biocel.2015.04.014 25966912

[23] Tang WW, Dietmann S, Irie N, Leitch HG, Floros VI, Bradshaw CR, et al Unique gene regulatory network resets the human germline epigenome for development. Cell. 2015; 161: 1453–1467. 10.1016/j.cell.2015.04.053 26046444 PMC4459712

[24] Sun D, Xi Y, Rodriguez B, Park HJ, Tong P, Meong M, et al MOABS: model based analysis of bisulfite sequencing data. Genome Biol. 2014; 15: R38 10.1186/gb-2014-15-2-r38 24565500 PMC4054608

[25] Sakaue M, Ohta H, Kumaki Y, Oda M, Sakaide Y, Matsuoka C, et al DNA methylation is dispensable for the growth and survival of the extraembryonic lineages. Curr Biol. 2010; 20: 1452–1457. 10.1016/j.cub.2010.06.050 20637626

[26] Lohmann F, Loureiro J, Su H, Fang Q, Lei H, Lewis T, et al KMT1E mediated H3K9 methylation is required for the maintenance of embryonic stem cells by repressing trophectoderm differentiation. Stem Cells. 2010; 28: 201–212. 10.1002/stem.278 20014010

[27] Hargan-Calvopina J, Taylor S, Cook H, Hu Z, Lee SA, Yen MR, et al Stage-specific demethylation in primordial germ cells safeguards against precocious differentiation. Dev Cell. 2016; 39: 75–86. 10.1016/j.devcel.2016.07.019 27618282 PMC5064860

[28] Liu S, Brind'Amour J, Karimi MM, Shirane K, Bogutz A, Lefebvre L, et al Setdb1 is required for germline development and silencing of H3K9me3-marked endogenous retroviruses in primordial germ cells. Genes Dev. 2014; 28: 2041–2055. 10.1101/gad.244848.114 25228647 PMC4173156

[29] Trapnell C, Pachter L, Salzberg SL. TopHat: discovering splice junctions with RNA-Seq. Bioinformatics. 2009; 25: 1105–1111. 10.1093/bioinformatics/btp120 19289445 PMC2672628

[30] Trapnell C, Roberts A, Goff L, Pertea G, Kim D, Kelley DR, et al Differential gene and transcript expression analysis of RNA-seq experiments with TopHat and Cufflinks. Nat Protoc. 2012; 7: 562–578. 10.1038/nprot.2012.016 22383036 PMC3334321

[31] Nimura K, Ishida C, Koriyama H, Hata K, Yamanaka S, Li E, Ura K, Kaneda Y. Dnmt3a2 targets endogenous Dnmt3L to ES cell chromatin and induces regional DNA methylation. Genes Cells. 2006;11: 1225–1237. 10.1111/j.1365-2443.2006.01012.x 16999741

[32] Kimura H, Hayashi-Takanaka Y, Goto Y, Takizawa N, Nozaki N. The organization of Histone H3 modifications as revealed by a panel of specific monoclonal antibodies. Cell Struct Funct. 2008; 33: 61–73. 18227620 10.1247/csf.07035

[33] Zeng PY, Vakoc CR, Chen ZC, Blobel GA, Berger SL. In vivo dual cross-linking for identification of indirect DNA-associated proteins by chromatin immunoprecipitation. Biotechniques. 2006; 41: 694–698. 10.2144/000112297 17191611

[34] Maatouk DM, Kellam LD, Mann MR, Lei H, Li E, Bartolomei MS, et al DNA methylation is a primary mechanism for silencing postmigratory primordial germ cell genes in both germ cell and somatic cell lineages. Development. 2006; 133: 3411–3418. 10.1242/dev.02500 16887828

[35] Hackett JA, Reddington JP, Nestor CE, Dunican DS, Branco MR, Reichmann J, et al Promoter DNA methylation couples genome-defence mechanisms to epigenetic reprogramming in the mouse germline. Development. 2012; 139: 3623–3632. 10.1242/dev.081661 22949617 PMC3436114

[36] Koike T, Wakai T, Jincho Y, Sakashita A, Kobayashi H, Mizutani E, et al DNA Methylation errors in cloned mouse sperm by germ line barrier evasion. Biol Reprod. 2016; 94: 1–7. 10.1095/biolreprod.116.138677 27103445

[37] Matsumura Y, Nakaki R, Inagaki T, Yoshida A, Kano Y, Kimura H, et al H3K4/H3K9me3 bivalent chromatin domains targeted by lineage-specific DNA methylation pauses adipocyte differentiation. Mol Cell. 2015; 60: 584–596 (2015). 10.1016/j.molcel.2015.10.025 26590716

[38] Bilodeau S, Kagey MH, Frampton GM, Rahl PB, Young RA. SetDB1 contributes to repression of genes encoding developmental regulators and maintenance of ES cell state. Genes Dev. 2009; 23: 2484–2489. 10.1101/gad.1837309 19884255 PMC2779743

[39] Sánchez C, Sánchez I, Demmers JA, Rodriguez P, Strouboulis J, Vidal M. Proteomics analysis of Ring1B/Rnf2 interactors identifies a novel complex with the Fbxl10/Jhdm1B histone demethylase and the Bcl6 interacting corepressor. Mol Cell proteomics. 2007; 6: 820–834. 10.1074/mcp.M600275-MCP200 17296600

[40] Bahar Halpern K, Vana T, Walker MD. Paradoxical role of DNA methylation in activation of FoxA2 gene expression during endoderm development. J Biol Chem. 2014; 289: 23882–23892. 10.1074/jbc.M114.573469 25016019 PMC4156079

[41] Voigt P, Tee WW, Reinberg D. A double take on bivalent promoters. Genes Dev. 2013; 27: 1318–1338. 10.1101/gad.219626.113 23788621 PMC3701188

[42] Mikkelsen TS, Ku M, Jaffe DB, Issac B, Lieberman E, Giannoukos G, et al Genome-wide maps of chromatin state in pluripotent and lineage-committed cells. Nature. 2007; 448: 553–560. 10.1038/nature06008 17603471 PMC2921165

[43] Sachs M, Onodera C, Blaschke K, Ebata KT, Song JS, Ramalho-Santos M. Bivalent chromatin marks developmental regulatory genes in the mouse embryonic germline in vivo. Cell Rep. 2013; 3: 1777–1784. 10.1016/j.celrep.2013.04.032 23727241 PMC3700580

[44] Karimi MM, Goyal P, Maksakova IA, Bilenky M, Leung D, Tang JX, et al DNA methylation and SETDB1/H3K9me3 regulate predominantly distinct sets of genes, retroelements, and chimeric transcripts in mescs. Cell Stem Cell 2011; 8: 676–687. 10.1016/j.stem.2011.04.004 21624812 PMC3857791

[45] Li Z, Dai H, Martos SN, Xu B, Gao Y, Li T, et al Distinct roles of DNMT1-dependent and DNMT1-independent methylation patterns in the genome of mouse embryonic stem cells. Genome Biol. 2015; 16: 115 10.1186/s13059-015-0685-2 26032981 PMC4474455

[46] Zhang T, Termanis A, Özkan B, Bao XX, Culley J, de Lima Alves F, et al G9a/GLP complex maintains imprinted DNA methylation in embryonic stem cells. Cell Rep. 2016; 15: 77–85. 10.1016/j.celrep.2016.03.007 27052169 PMC4826439

[47] Matsui T, Leung D, Miyashita H, Maksakova IA, Miyachi H, Kimura H, et al Proviral silencing in embryonic stem cells requires the histone methyltransferase ESET. Nature. 2010; 464: 927–931. 10.1038/nature08858 20164836

[48] Yang BX, El Farran CA, Guo HC, Yu T, Fang HT, Wang HF, et al Systematic identification of factors for provirus silencing in embryonic stem cells. Cell. 2015; 163: 230–245. 10.1016/j.cell.2015.08.037 26365490 PMC4686136

[49] Maksakova IA, Thompson PJ, Goyal P, Jones SJ, Singh PB, Karimi MM, Lorincz MC. Distinct roles of KAP1, HP1 and G9a/GLP in silencing of the two-cell-specific retrotransposon MERVL in mouse ES cells. Epigenetics Chromatin. 2013; 6: 15 10.1186/1756-8935-6-15 23735015 PMC3682905

