# Supplementary figures and images for: DNMTs and SETDB1 function as co-repressors in MAX-mediated repression of germ cell–related genes in mouse embryonic stem cells
Source: PLoS One. 2018 Nov 7;13(11):e0205969. doi: 10.1371/journal.pone.0205969 (PMC6221296; doi:10.1371/journal.pone.0205969)

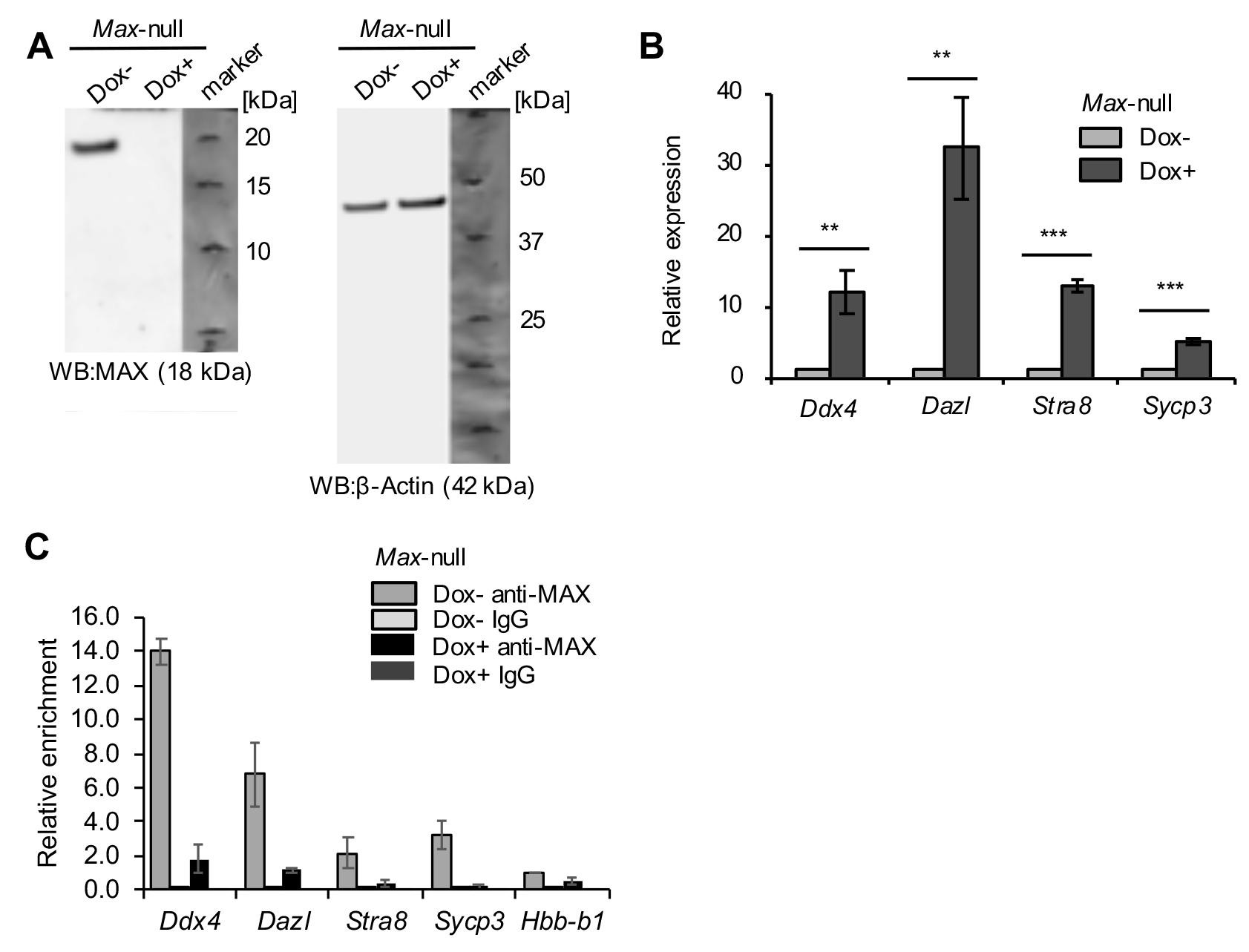

Supplement: S1 Fig — (A) Dox-dependent attenuation of MAX protein levels in Max-null ESCs was assessed by Western blotting using anti-MAX antibody. Principally, the same result was obtained in two independent experiments. (B) Relative expression of the late PGC markers in Max-null ESCs (Dox+), as determined by qRT-PCR. The expression in control ESCs (Dox−) was set as 1.0. Values are plotted as the mean ± SEM of 3 biological replicates. **P < 0.01, ***P < 0.001 (Student’s t-test). (C) ChIP-qPCR analyses of Max-null ESCs (Dox+) and control ESCs (Dox−) using anti-MAX antibody or control IgG for the promoter region of the late PGC markers and hemoglobin β (Hbb-b1) as a negative control of MAX localization. The data are displayed in the same way as in Fig 1A. (TIF) [file pone.0205969.s001.tif]

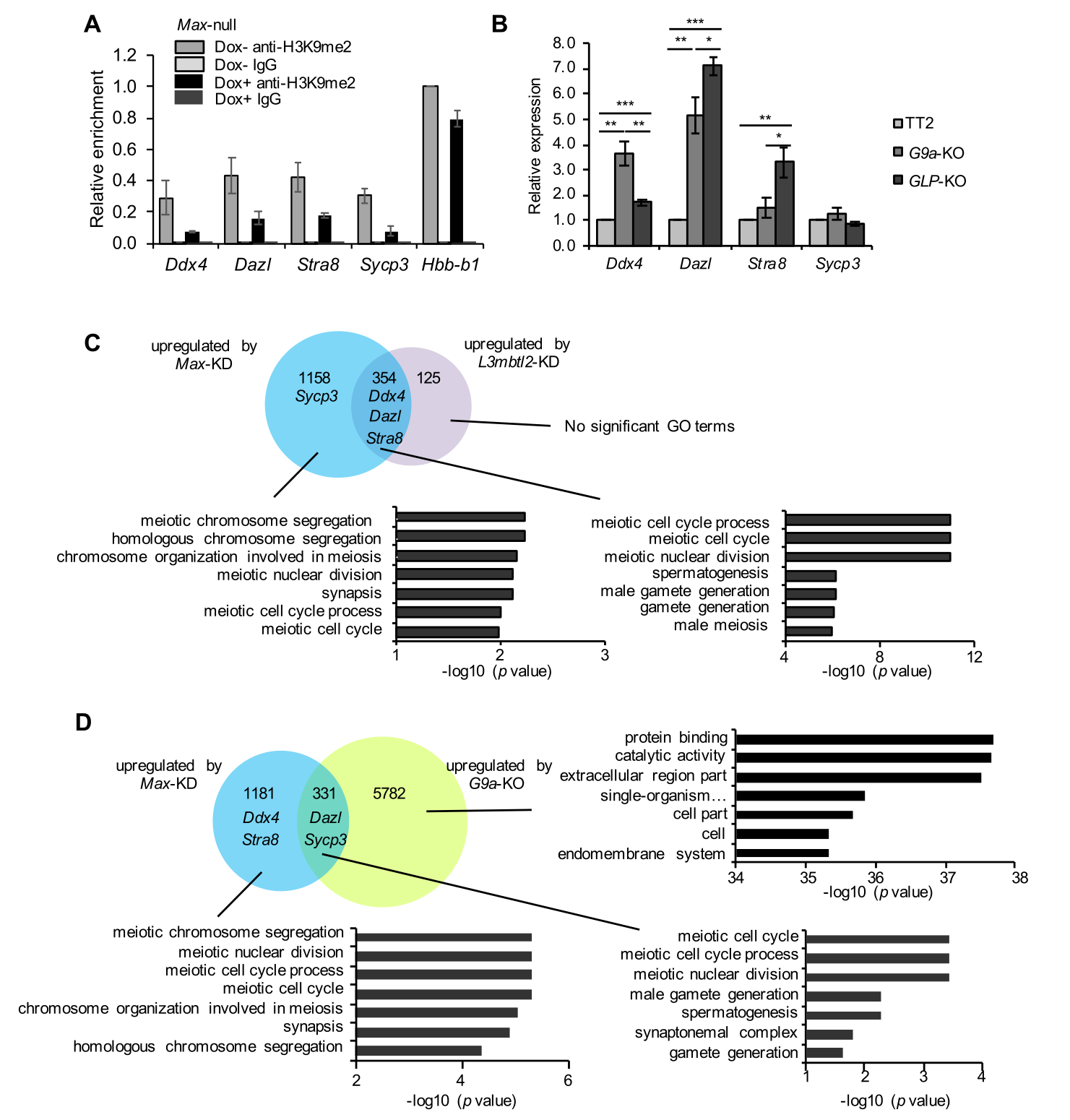

Supplement: S2 Fig — (A) ChIP-qPCR analyses of Max-null ESCs (Dox+) and control ESCs (Dox−) using anti-H3K9me2 antibody or control IgG. The data are displayed in the same way as in Fig 1A. (B) Relative expression of the late PGC markers in G9a- or GLP-KO ESCs as determined by qRT-PCR. The expression in control ESCs (TT2) was set as 1.0. Values are plotted as the mean ± SEM of 3 biological replicates. *P < 0.05, **P < 0.01, ***P < 0.001 (Student’s t-test). (C to F) Venn diagram of genes up-regulated in Max-KD ESCs (GSE45181) [2] (n = 3, > 2-fold change, one-way ANOVA P < 0.05) compared with genes up-regulated in L3mbtl2-KD ESCs (n = 4, > 2-fold change, one-way ANOVA P < 0.05) (C), G9a-KO ESCs (n = 1, > 1.3-fold change) (D), Ring1a/b-DKO ESCs (GSE10573) [16] (n = 1, > 1.5-fold change) (E), or Pcgf6-KO ESCs (GSE84480) [13] (n = 2, > 2-fold change) (F). GO analyses of genes representing each category were performed. GO terms with the lowest corrected P value (top 7) are shown. (TIF) [file pone.0205969.s002.tif]

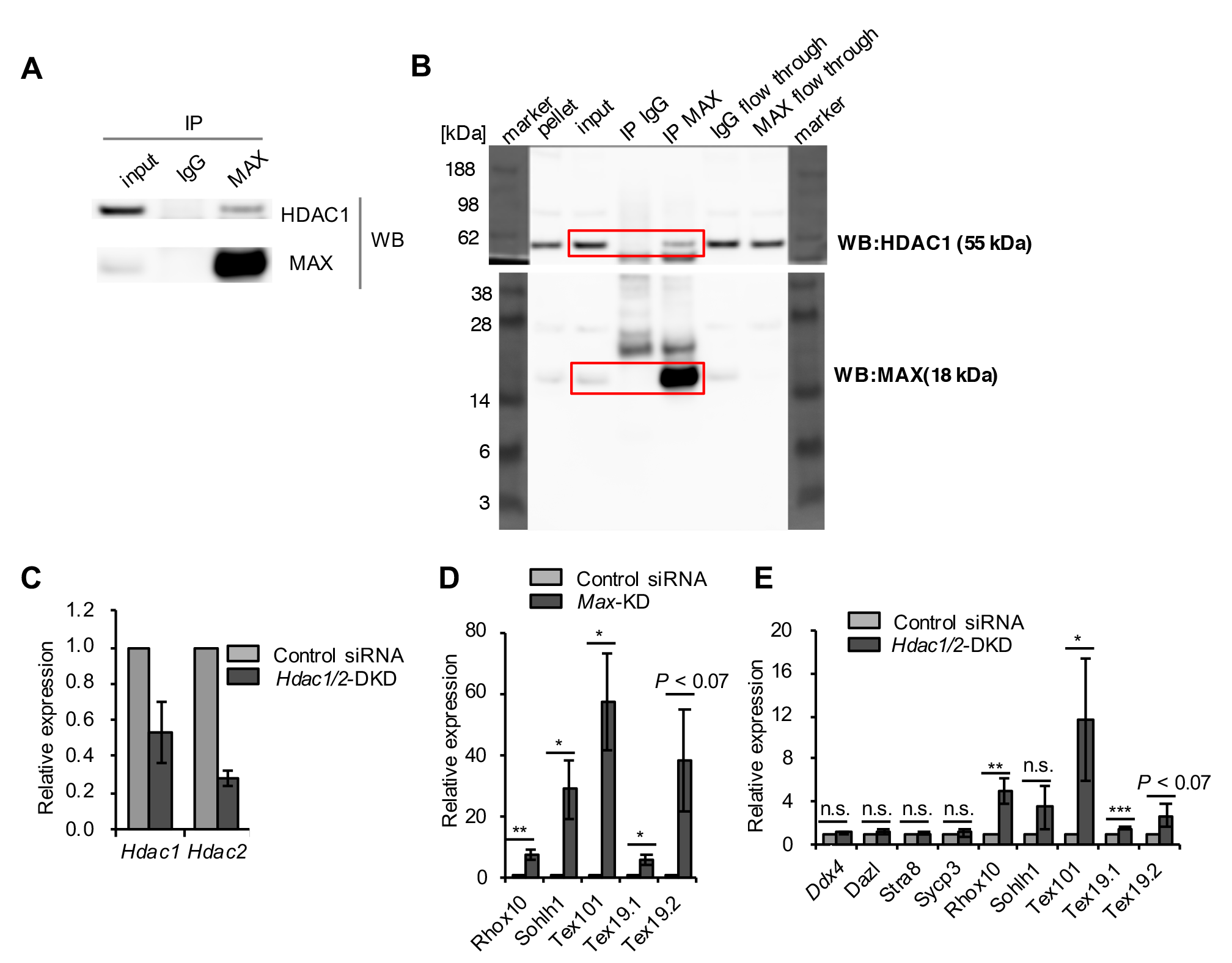

Supplement: S3 Fig — (A) Immunoprecipitated samples using anti-MAX antibody or control IgG were analyzed by Western blotting using anti-HDAC1 antibody. Principally, the same result was obtained in two independent experiments. (B) Un-cropped data of Western blotting corresponding to S3A Fig. Immunoprecipitated samples by anti-MAX antibody or control IgG were subjected to Western blotting by using anti-HDAC1 antibody. Red indicates the data shown in S3A Fig. (C) KD efficiency of Hdac1 and Hdac2 in ESCs at day 2 post-siRNA treatment. (D) Relative expression of germ cell-related genes (Rhox10, Sohlh2, Tex101, Tex19.1, and Tex19.2) in Max-KD ESCs at day 3 post-siRNA treatment (4 biological replicates), as determined by qRT-PCR. (E) Relative expression of the late PGC marker genes, Rhox10, Sohlh2, Tex101, Tex19.1, and Tex19.2 in Hdac1/2-DKD ESCs at day 3 post-siRNA treatment (3 biological replicates), as determined by qRT-PCR. The expression in control ESCs was set as 1.0. Values are plotted as the mean ± SEM. n.s; not significant, *P < 0.05, **P < 0.01, ***P < 0.001 (Student’s t-test). (TIF) [file pone.0205969.s003.tif]

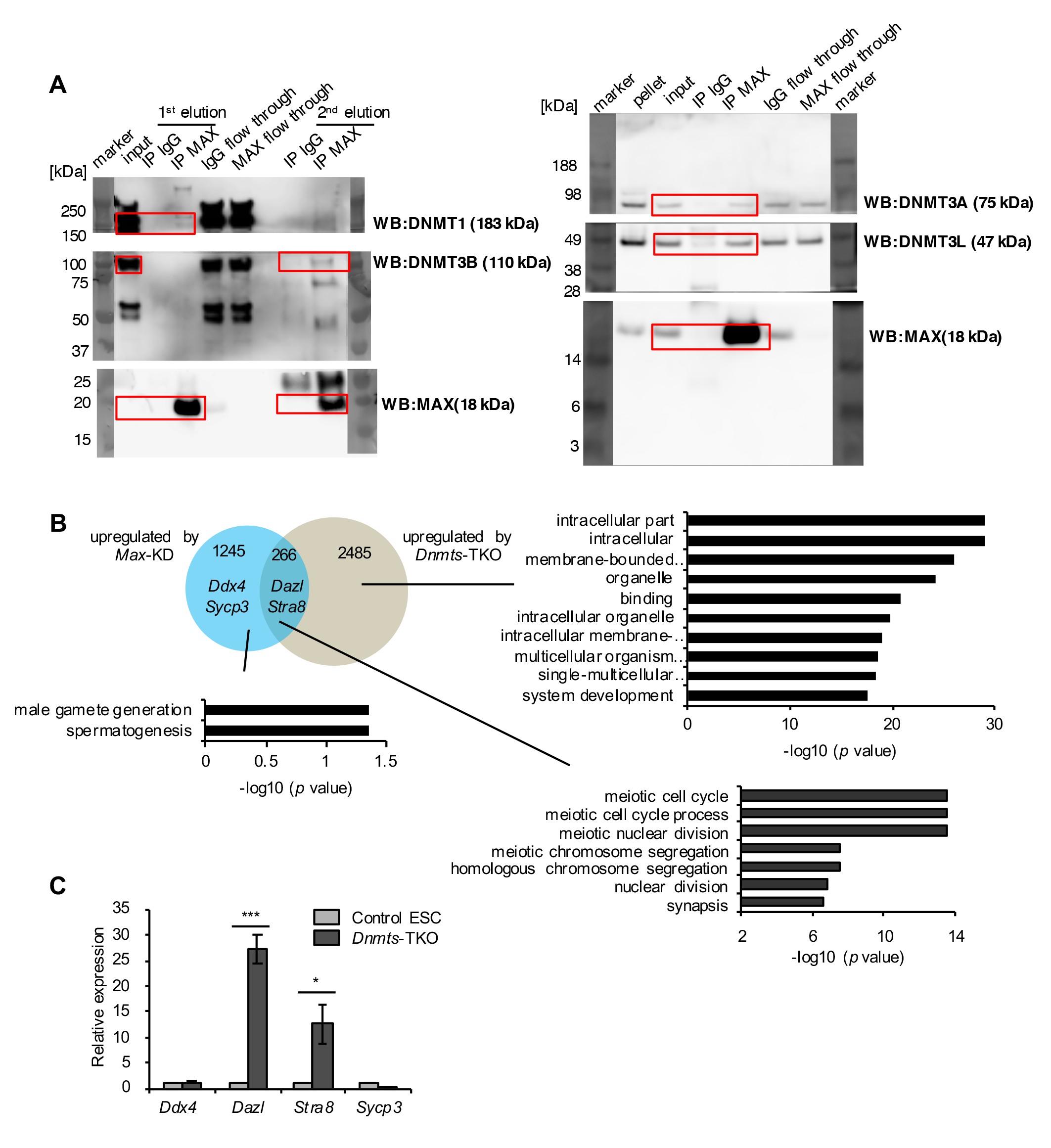

Supplement: S4 Fig — (A) Un-cropped data of Western blotting corresponding to Fig 2A. Immunoprecipitated samples by anti-MAX antibody or control IgG were subjected to Western blotting by using anti-DNMT antibodies. Red indicates the data shown in Fig 2A. The elution was performed twice and each eluted sample was analyzed separately. (B) Venn diagram of genes up-regulated in Max-KD ESCs (GSE45181) [2] and Dnmts-TKO ESCs (GSE20177) [25] (Max-KD ESCs; n = 3, > 2-fold change, one-way ANOVA P < 0.05, Dnmts-TKO ESCs; n = 2, > 1.3-fold change). GO analyses of genes representing each category were performed. GO terms with the lowest corrected P value (top 7) are shown. (C) Relative expression of the late PGC markers in Dnmts-TKO ESCs determined by qRT-PCR. The expression in control ESCs was set as 1.0. Values are plotted as the mean ± SEM of 3 biological replicates. *P < 0.05, ***P < 0.001 (Student’s t-test). (TIF) [file pone.0205969.s004.tif]

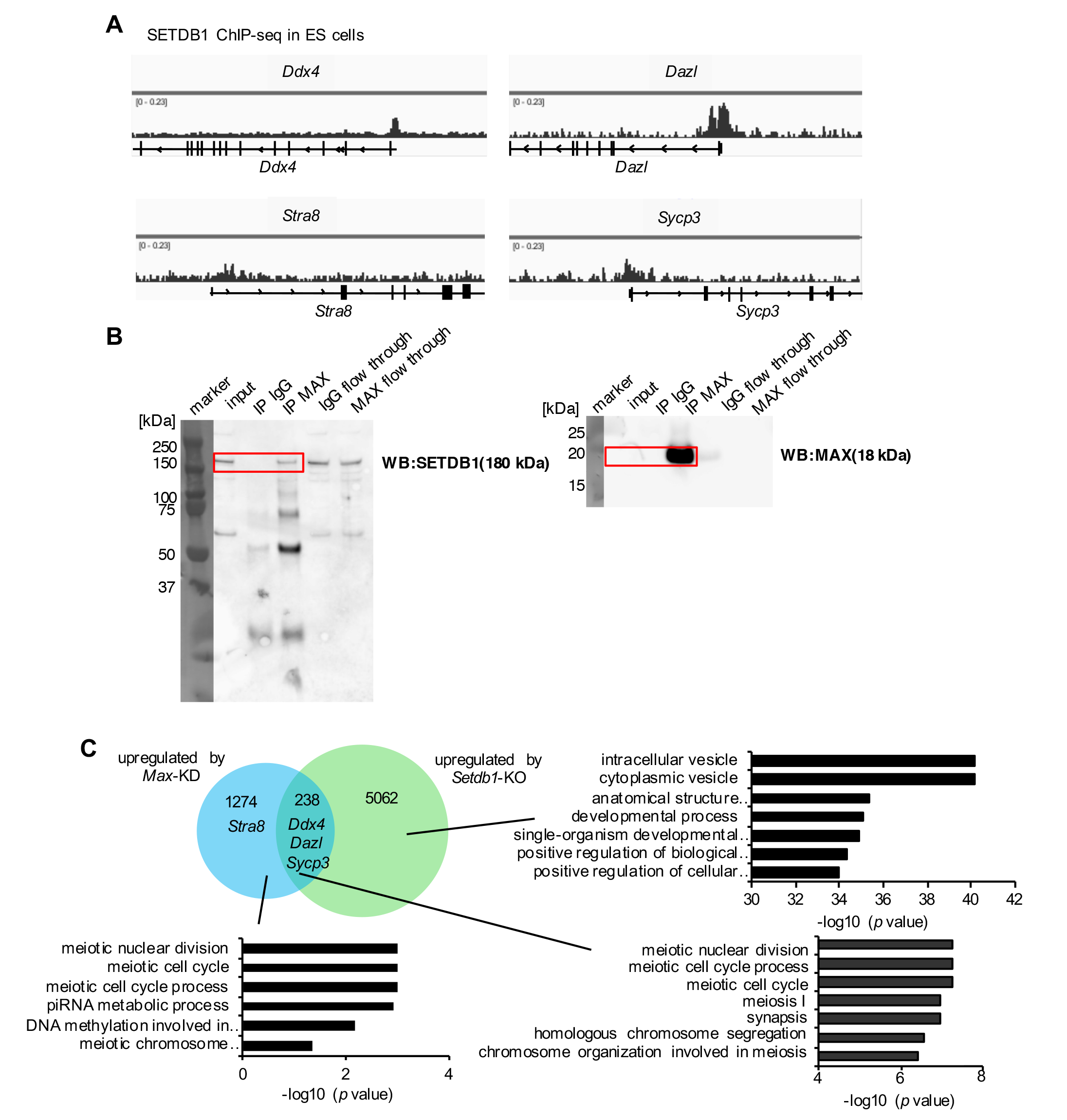

Supplement: S5 Fig — (A) ChIP-seq data for SETDB1 in ESCs (GSE73434) [37] were re-analyzed using Integrative Genomics Viewer (IGV). Neighboring regions of TSSs of the late PGC markers are shown. (B) Un-cropped data of Western blotting corresponding to Fig 3A. Immunoprecipitated samples by anti-MAX antibody or control IgG were subjected to Western blotting by using anti-SETDB1 antibody. Red indicates the data shown in Fig 3A. (C) Venn diagram of genes up-regulated in Max-KD ESCs (GSE45181) [2] and Setdb1-KO ESCs (GSE28593) [26] (Max-KD ESCs; n = 3, > 2-fold change, one-way ANOVA P < 0.05, Setdb1-KO ESCs; n = 3, > 1.3-fold change, one-way ANOVA P < 0.05). GO analyses of genes representing each category were performed. GO terms with the lowest corrected P value (top 7) are shown. (TIF) [file pone.0205969.s005.tif]

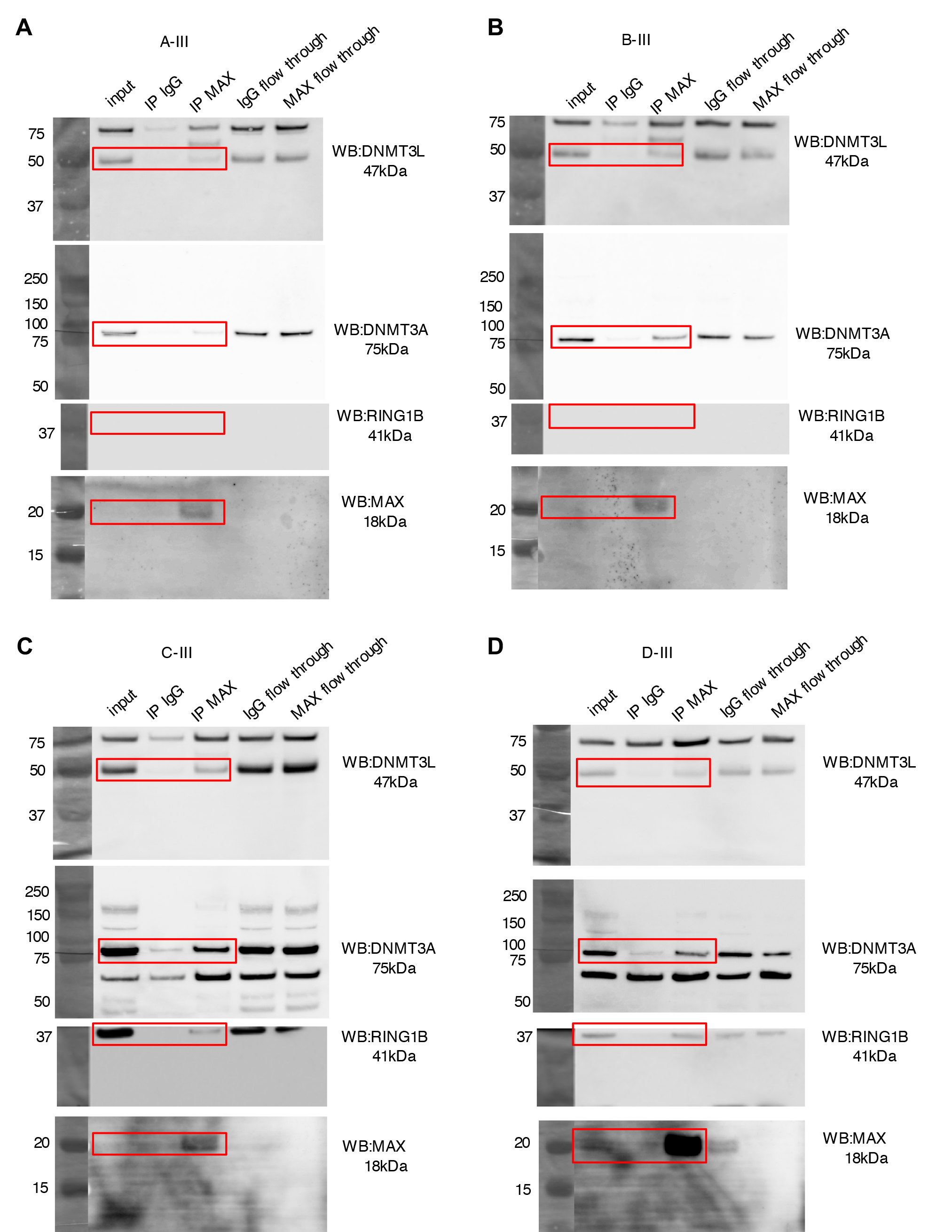

Supplement: S6 Fig — (A to D) Un-cropped data of Western blotting corresponding to Fig 5B–5E, respectively. Immunoprecipitated samples by anti-MAX antibody or control IgG were subjected to Western blotting by using anti-DNMT3A, DNMT3L, RING1B antibodies for fraction A-III (A), B-III (B), C-III (C), or D-III (D). Red indicates the data shown in Fig 5B–5E, respectively. (TIF) [file pone.0205969.s006.tif]

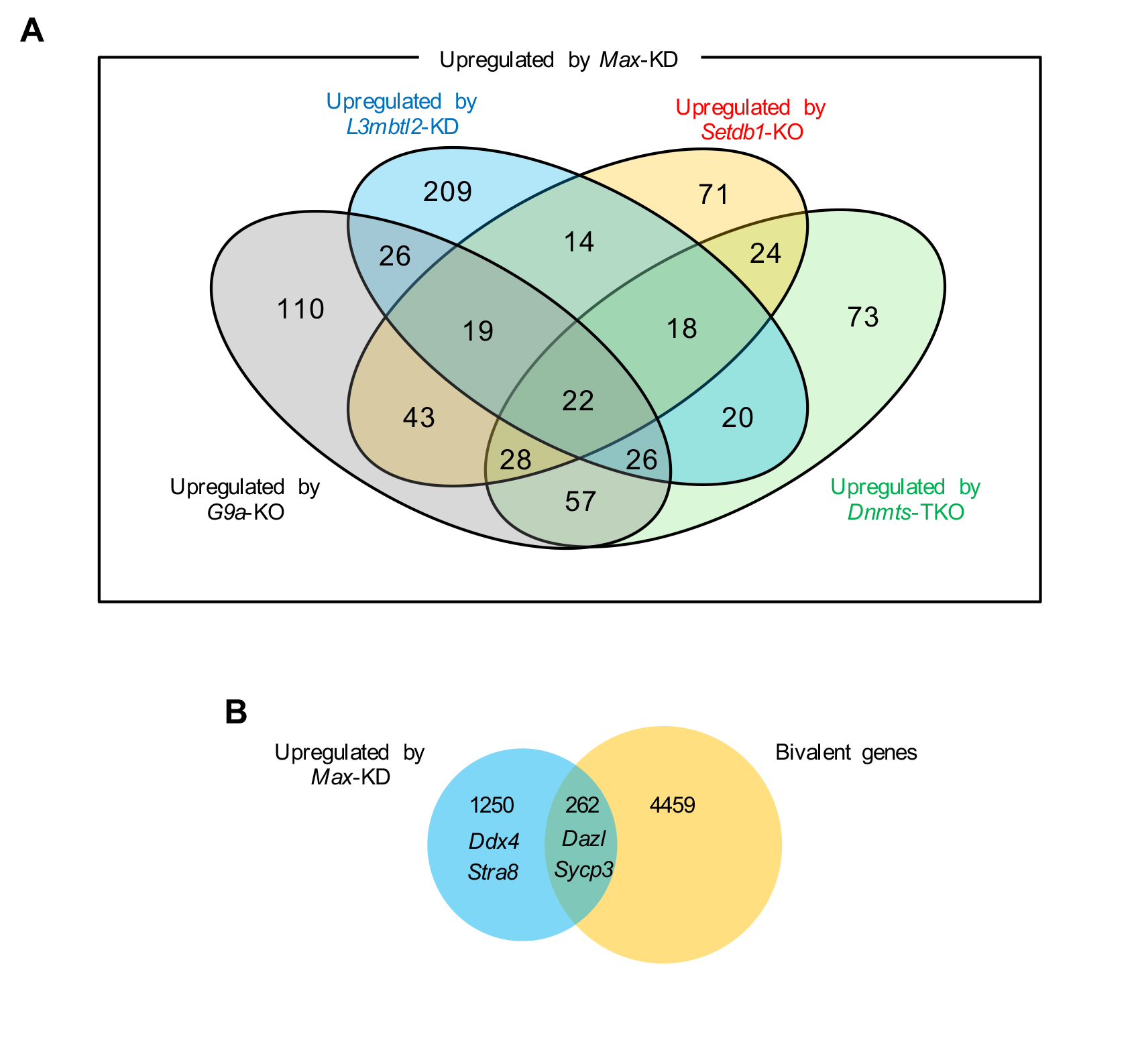

Supplement: S7 Fig — (A) Venn diagram of genes up-regulated in L3mbtl2-KD ESCs (n = 4, > 2-fold change, one-way ANOVA P < 0.05), G9a-KO ESCs (n = 1, > 1.3-fold change), Setdb1-KO ESCs (GSE28593) [26] (n = 3, > 1.3-fold change, one-way ANOVA P < 0.05), and Dnmts-TKO ESCs (GSE20177) [25] (n = 2, > 1.3-fold change) among up-regulated genes in Max-KD ESCs (GSE45181) [2] (n = 3, > 2-fold change, one-way ANOVA P < 0.05). (B) Venn diagram showing relationships between genes up-regulated in Max-KD ESCs [2] and the bivalent genes [42,43]. (TIF) [file pone.0205969.s007.tif]

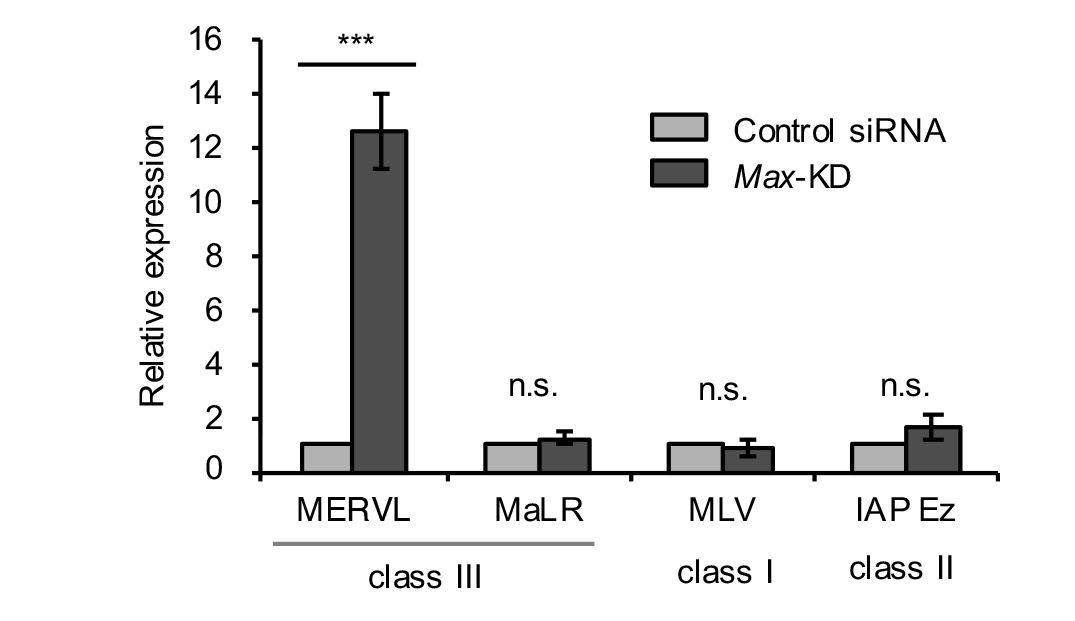

Supplement: S8 Fig — Relative expression of class I-III ERVs in Max-KD ESCs, as determined by qRT-PCR. The expression in VV3 ESCs treated with control siRNA was set as 1.0. Values are plotted as the mean ± SEM of 3 biological replicates. n.s.: not significant, ***P < 0.001 (Student’s t-test). (TIF) [file pone.0205969.s008.tif]

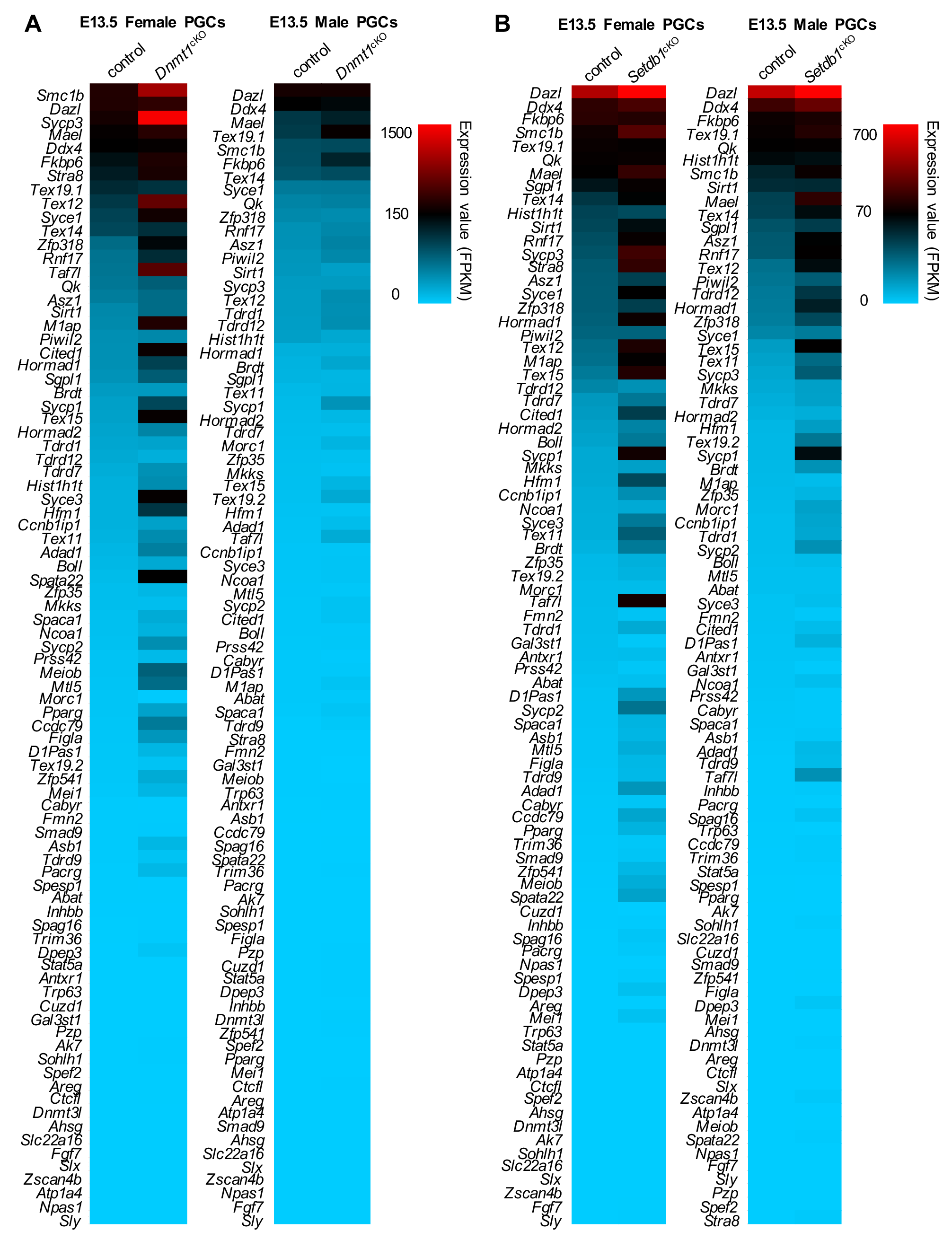

Supplement: S9 Fig — (A and B) Germ cell–related genes up-regulated in Max-KD ESCs compared with control ESCs (85 genes, n = 3, > 2-fold change, ANOVA P < 0.05, with GO term “reproduction”, S3 Table) were selected and expression change of these genes in E13.5 Setdb1cKO PGCs (GSE60377) [28] (n = 2) (A) or Dnmt1cKO PGCs (GSE74938) [27] (n = 3) (B) compared with control PGCs were represented as heat maps. (TIF) [file pone.0205969.s009.tif]

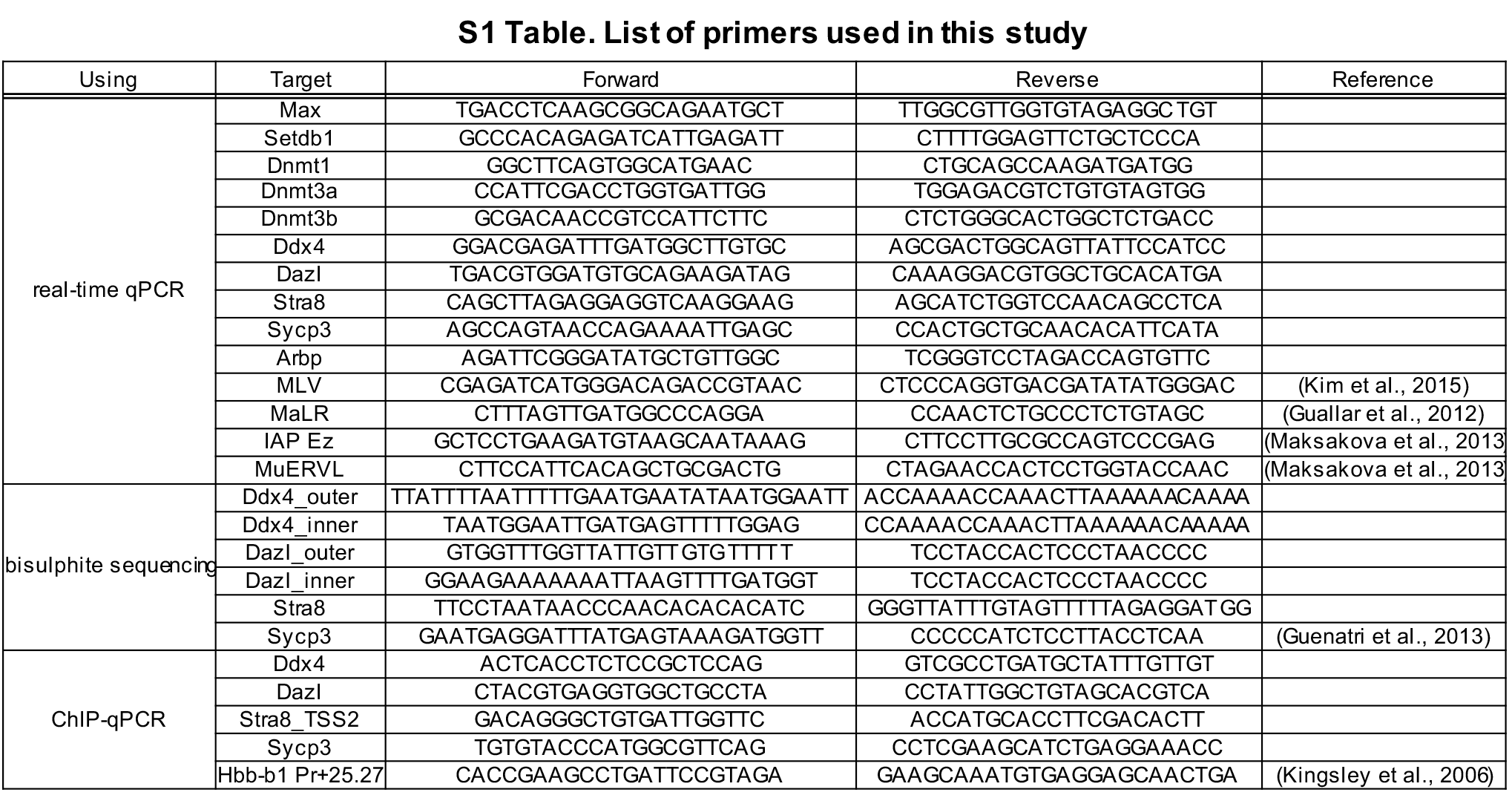

Supplement: S1 Table — (TIF) [file pone.0205969.s010.tif]

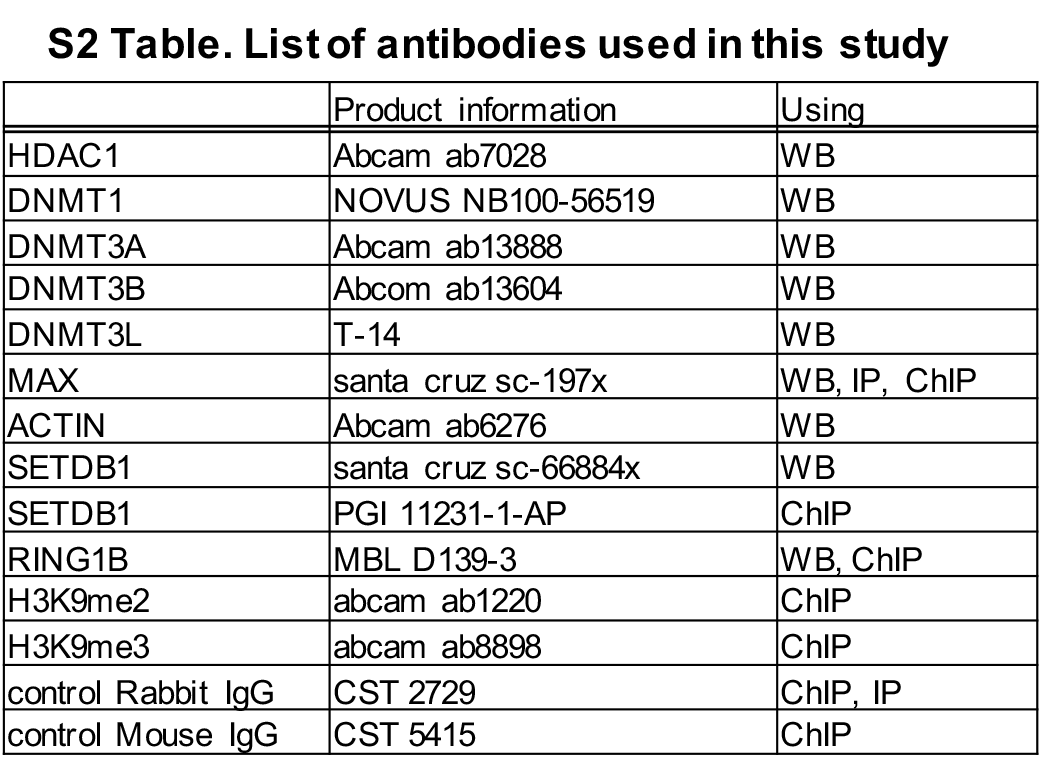

Supplement: S2 Table — (TIF) [file pone.0205969.s011.tif]

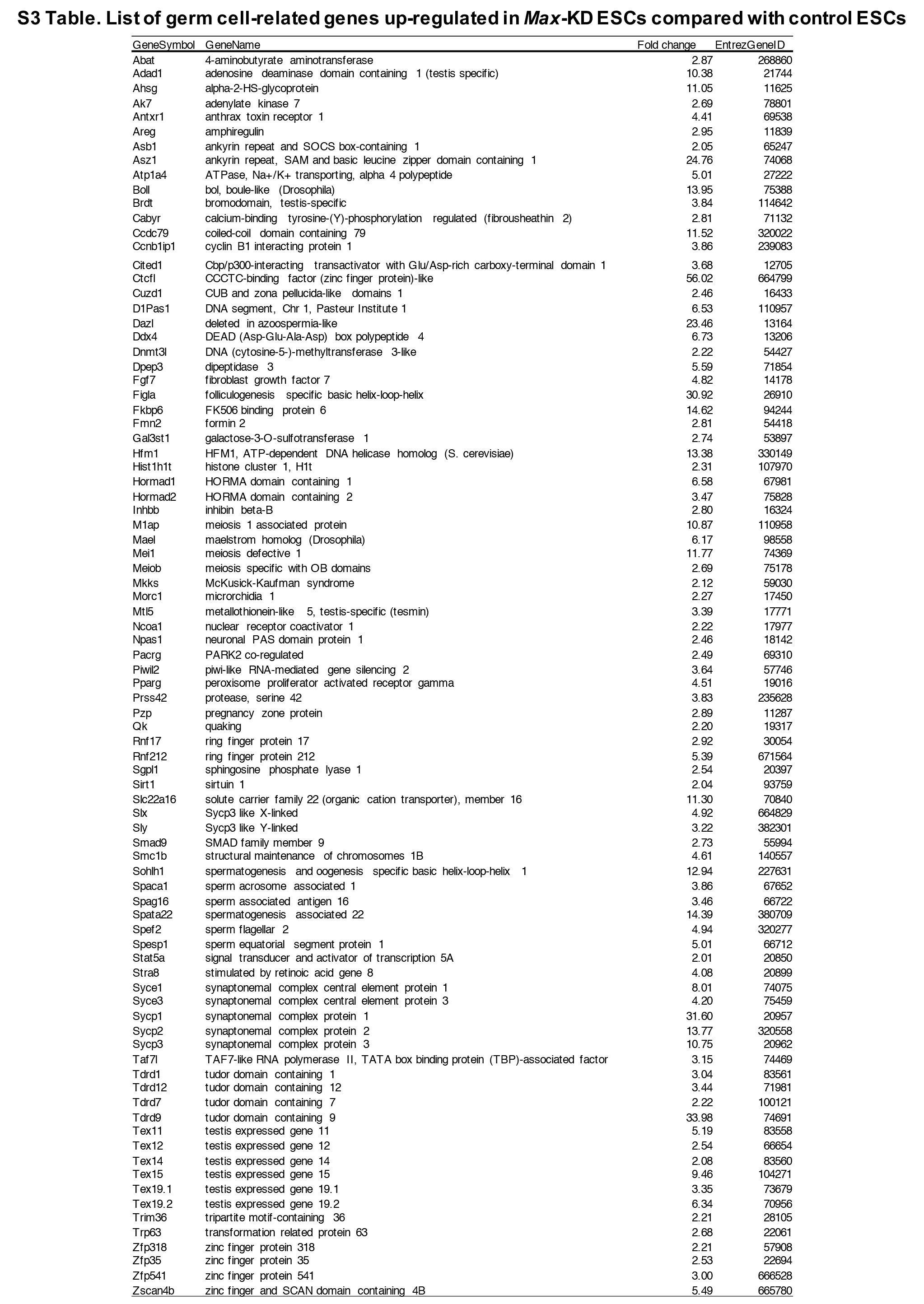

Supplement: S3 Table — Eighty-five of germ cell–related genes with gene ontology term “reproduction” were extracted as up-regulated genes in Max-KD ESCs compared with control ESCs (GSE45181) [2] (n = 3, > 2-fold change, one-way analysis of variance [ANOVA] P < 0.05). (TIF) [file pone.0205969.s012.tif]

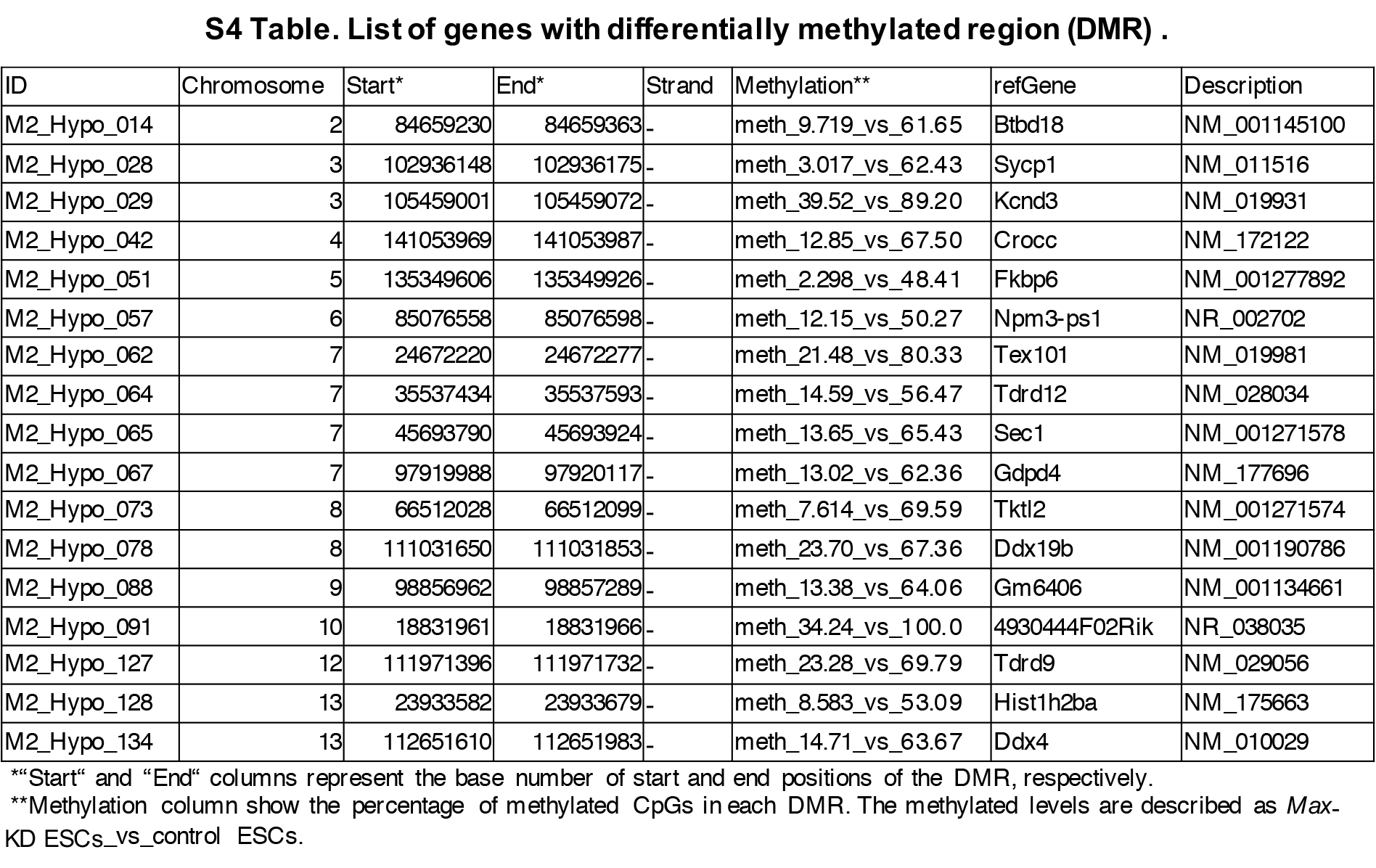

Supplement: S4 Table — (TIF) [file pone.0205969.s013.tif]

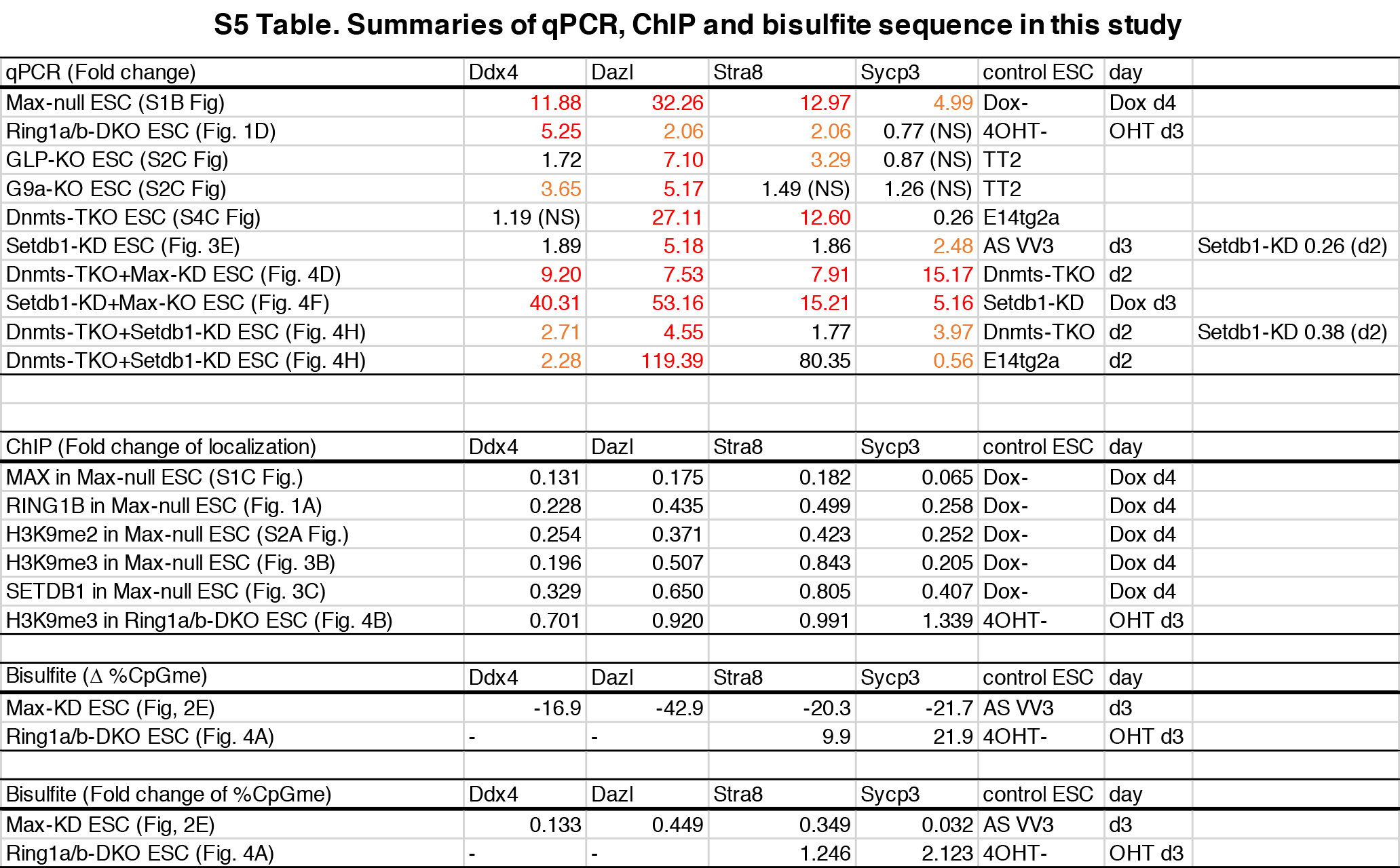

Supplement: S5 Table — Red and orange indicate > 5 fold and > 2 fold up-regulated genes in RT-qPCR, respectively. (TIF) [file pone.0205969.s014.tif]
